# Single-Cell RNA Sequencing Reveals Cellular and Transcriptional Changes Associated With M1 Macrophage Polarization in Hidradenitis Suppurativa

**DOI:** 10.3389/fmed.2021.665873

**Published:** 2021-08-24

**Authors:** Paula Mariottoni, Simon W. Jiang, Courtney A. Prestwood, Vaibhav Jain, Jutamas Suwanpradid, Melodi Javid Whitley, Margaret Coates, David A. Brown, Detlev Erdmann, David L. Corcoran, Simon G. Gregory, Tarannum Jaleel, Jennifer Y. Zhang, Tamia A. Harris-Tryon, Amanda S. MacLeod

**Affiliations:** ^1^Department of Dermatology, School of Medicine, Duke University, Durham, NC, United States; ^2^Department of Dermatology, University of Texas Southwestern Medical Center, Dallas, TX, United States; ^3^Duke Molecular Physiology Institute, Duke University, Durham, NC, United States; ^4^Division of Plastic, Maxillofacial, and Oral Surgery, Duke University Medical Center, Durham, NC, United States; ^5^Duke Center for Genomic and Computational Biology, Duke University, Durham, NC, United States; ^6^Department of Neurology, Duke University School of Medicine, Durham, NC, United States; ^7^Department of Immunology, University of Texas Southwestern Medical Center, Dallas, TX, United States; ^8^Department of Immunology, Duke University, Durham, NC, United States; ^9^Department of Molecular Genetics and Microbiology, Duke University, Durham, NC, United States

**Keywords:** single cell sequencing, macrophage-cell, hidradenitis suppurativa, antiviral immune pathways, non-healing wounds, interferon

## Abstract

Hidradenitis suppurativa (HS) is a chronic inflammatory skin disease characterized by recurrent abscesses, nodules, and sinus tracts in areas of high hair follicle and sweat gland density. These sinus tracts can present with purulent drainage and scar formation. Dysregulation of multiple immune pathways drives the complexity of HS pathogenesis and may account for the heterogeneity of treatment response in HS patients. Using transcriptomic approaches, including single-cell sequencing and protein analysis, we here characterize the innate inflammatory landscape of HS lesions. We identified a shared upregulation of genes involved in interferon (IFN) and antimicrobial defense signaling through transcriptomic overlap analysis of differentially expressed genes (DEGs) in datasets from HS skin, diabetic foot ulcers (DFUs), and the inflammatory stage of normal healing wounds. Overlap analysis between HS- and DFU-specific DEGs revealed an enrichment of gene signatures associated with monocyte/macrophage functions. Single-cell RNA sequencing further revealed monocytes/macrophages with polarization toward a pro-inflammatory M1-like phenotype and increased effector function, including antiviral immunity, phagocytosis, respiratory burst, and antibody-dependent cellular cytotoxicity. Specifically, we identified the STAT1/IFN-signaling axis and the associated IFN-stimulated genes as central players in monocyte/macrophage dysregulation. Our data indicate that monocytes/macrophages are a potential pivotal player in HS pathogenesis and their pathways may serve as therapeutic targets and biomarkers in HS treatment.

## Introduction

Hidradenitis suppurativa (HS) is a chronic inflammatory skin disease with a relapsing and remitting course. The disorder is characterized by painful abscesses and nodules in areas with high apocrine sweat gland (SG) and hair follicle density, such as the groin, axilla, and buttocks (1). Severe features of HS disease may include sinus tracts, malodorous discharge, and extensive scarring in affected skin areas (1). These debilitating features can cause substantial psychosocial burden, increasing the risk of mood disorders and completed suicide (2, 3).

Despite the significant physical and psychosocial morbidity associated with the diagnosis, the pathogenesis of HS still remains incompletely understood. An interplay of multiple environmental factors, genetics, host-microbe interactions, and immune dysregulation likely contribute to disease risk and severity (1). The underlying mechanisms of immune dysregulation are especially complex and interesting, as multiple pathways in innate and adaptive immunity are involved. Across multiple studies, tumor necrosis factor (TNF)-α, interleukin (IL)-1β, IL-12, IL-17, IL-23, and complement have been identified as relevant targets for immunotherapy in patients with HS (4–8).

Macrophages secrete the cytokines TNF-α, IL-1β, and IL-23 and are among the most abundant cell types found in HS lesions (9, 10). Notably, macrophages producing TNF-α and IL-1β are characteristic of “pro-inflammatory” and “phagocytic” M1-like macrophages (11). M1 macrophages are typically induced by interferon (IFN) and microbial signals, such as lipopolysaccharide (LPS) (12). A phenotypic switch from proinflammatory M1 macrophages to anti-inflammatory M2 macrophages is a key step in the transition from the inflammatory phase of wound healing to the proliferative phase, as well as for the resolution of various inflammatory skin diseases (13, 14). Dysregulation of this switch can perturb the inflammatory phase and lead to chronic non-healing wounds, as found in diabetic foot ulcers (DFUs) and chronic venous leg ulcers (14). The M1 to M2 macrophage transition may also be of particular relevance in understanding the disease mechanism of HS, which is characterized by aberrant skin inflammation and tissue disorganization (15).

In addition to infiltration of macrophages and other immune cells, HS skin is characterized by high expression of antimicrobial peptides and proteins (AMPs) and antiviral proteins (AVPs) (16). Transcriptomic analysis from our group has revealed the upregulation of many genes encoding for AMPs and AVPs, including S100 family proteins, human β-defensin (hBD)-2, and oligoadenylate synthetase (OAS) proteins in HS lesions (16). These molecules are components of the early innate immune response and are typically activated in both keratinocytes and immune cells to destroy invasive microorganisms and protect the host barrier (17). In addition to their direct antimicrobial activity, AMPs also play immunomodulatory roles. For instance, cathelicidin LL-37 and hBD-2 regulate chemotaxis of immune cells, while LL-37 has been described to promote wound closure and re-epithelialization (17, 18). AMPs and AVPs can also serve as important biomarkers for the induction of specific immune pathways. Perhaps one of the most important of these pathways is IFN signaling, which canonically leads to activation of antiviral and antimicrobial immunity. OAS family proteins are part of the AVPs induced by type I IFNs that lead to downstream activation of the antiviral enzyme RNase L (19). Other AVPs are induced by type II (IFN-γ) and type III (IFN-λ) IFNs (20, 21). IFN-γ and LPS induce expression of the AMPs hBD-1 and hBD-2 in monocytes and monocyte-derived macrophages (22).

Though our previous work had shown the dysregulation of AMP and AVP expression in HS lesions, the transcriptomic data was unable to reveal the specific cell types that contribute to this dysregulation (16). Furthermore, numerous studies have found upregulation of type I and II IFN pathways in HS lesions (23–26). The current body of work is deficient in profiling of HS lesions at the single-cell level, which can elucidate the key cell types implicated in these responses and highlight the most relevant pathways. Additionally, our dataset uncovered a shared antimicrobial gene signature between HS skin and healing wounds (HW), but there are limited data comparing HS lesions and chronic, non-healing wounds (16). This latter comparison may elucidate additional features of the disease given that HS lesions share clinical features with chronic venous leg ulcers and DFUs including a tendency to form chronic open lesions, prolonged inflammatory responses, and increased gram negative microbiota (27–29). Thus, understanding the cell types and pathways involved in dysregulation of antiviral and antimicrobial responses is crucial to developing potential novel therapies for HS.

Our interest and earlier work in AMPs/AVPs and wound healing led us to characterize the cell types and innate immune pathways that may contribute to HS lesions. First, we analyzed and compared transcriptomic data from DFUs, HS lesions, and HWs to show that HS and chronic non-healing wounds are enriched in immune processes indicative of macrophage function. To provide insight into the transcriptomic landscape of non-healing HS lesions on a per-cell basis, we utilized single-cell RNA sequencing (scRNA-seq) technology. We demonstrate that innate immune processes are upregulated not only in immune cells, such as monocytes and macrophages, but also in keratinocytes and SG cells. Monocytes and macrophages are upregulated in genes indicative of polarization toward an M1 macrophage phenotype. These cells have increased expression of genes that function in phagocytosis, respiratory burst, antibody-dependent cellular toxicity (ADCC), and type I and type II IFN signaling. Multiple cell types have increased expression of key AMPs, such as S100 family proteins, as well as AVPs. With upregulated genes associated with activation of mononuclear phagocytes, effectors of ADCC, and IFN signaling, the transcriptional landscape of HS skin is skewed toward type 1 immunity. Together, our data show that HS skin possesses a proinflammatory transcriptional profile associated with M1-like macrophage polarization, which may exacerbate continued inflammation and impede wound healing.

## Methods

### Microarray and Bulk RNA-Sequencing Overlap Comparison

We used the publicly available microarray datasets from Blok et al. (30) (GSE72702) and Ramirez et al. (31) (GSE80178) as well as the RNA-seq data set from Iglesias-Bartolome et al. (32) (GSE97615). The data from each study were downloaded from the Gene Expression Omnibus (33).

The Blok et al. (30) (GSE72702) dataset contains mRNA microarray experiments performed on skin biopsy samples from patients with HS. The samples were divided between lesional skin (*N* = 17) and healthy skin (*N* = 13); all 13 healthy skin samples were paired with one lesional skin sample. We used the Robust Multichip Average normalized dataset provided by the authors as available in the Gene Expression Omnibus (33). The four lesional skin samples for which there were no matched healthy skin samples were removed from subsequent analysis. We first filtered lowly-expressed and invariant microarray probes, i.e., with an expression level ≥4 in <3 samples, or a standard deviation < 0.1. After filtering, the dataset consisted of 51,567 probes. To identify differentially expressed genes (DEGs) between lesional and non-lesional samples, we used the R package nlme to implement a mixed-effects model including the variable “patient” as a random effect (34). The Benjamini-Hochberg method was used to correct for multiple hypothesis testing.

The Ramirez et al. (31) (GSE80178) dataset contains mRNA microarray experiments carried out on DFU samples, diabetic foot skin, or non-diabetic foot skin. Data were normalized with the Robust Multichip Average preprocessing methodology using R package oligo to eliminate systematic differences across arrays (35). We mapped microarray probes (53,617) to gene names based on annotation from the hugene20sttranscriptcluster.db R package (36). Probes were excluded from the analysis if they did not match a known gene. This resulted in 29,208 remaining annotated probes for downstream analysis. Statistically significant DEGs were determined for each of the following phenotypic comparisons: “Diabetic Foot Ulcer vs. Diabetic Foot Skin” and “Diabetic Foot Ulcer vs. Foot Skin” using an empirical Bayes moderated test statistic from the limma R package (37). The *p*-values were corrected for multiple hypothesis testing by the Benjamini-Hochberg method. Genes were considered differentially-expressed in the foot ulcer if they had an adjusted *p*-value ≤ 0.05 in each comparison with the diabetic foot skin and the healthy foot skin.

The RNA-sequencing dataset from Iglesias-Bartolome et al. (32) (GSE97615) contains samples from human axillary skin wounds at baseline (Day 1, unwounded), 2 days after full-thickness 3-mm punch biopsy wounding (Day 3), and 5 days after wounding (Day 6). The raw data were processed using the fastp toolkit to trim low-quality bases and Illumina sequencing adapters from the 3′ end of the reads (38). Only reads that were 20 nucleotides or longer after trimming were kept for further analysis. Reads were mapped to the GRCh38v93 version of the human genome and transcriptome using the STAR RNA-seq alignment tool (39, 40). Reads were kept for subsequent analysis if they mapped to a single genomic location. Gene counts were compiled using the feature Counts tool (41). Only genes that had at least 10 reads in any given library were used for subsequent analysis. This resulted in a set of 12 samples and 23,378 genes. Normalization and differential expression between Day 6 and Day 1, as well as Day 3 and Day 1, were carried out using the DESeq2 Bioconductor package with the R statistical programming environment (42, 43). The *p*-values were corrected for multiple hypothesis testing by the Benjamini-Hochberg method. A gene was considered downregulated if it had an adjusted *p-*value ≤ 0.05 and a negative fold-change in either the Day 3-vs.-Day 1 analysis or the Day 6-vs.-Day 1 analysis. Likewise, a gene was considered upregulated if it was significant in either comparison along with a positive fold-change.

Genes were considered in the overlap analysis if they (1) had an adjusted *p*-value ≤ 0.05 in that particular dataset and (2) were changing in the same direction relative to their respective controls.

### Preparation of Single-cell Suspension

HS patients undergoing surgical excision for Hurley stage 3 therapy were consented to provide skin tissue for scRNA-seq analysis (Supplementary Table 1). Surgical excisions from 3 patients with HS (axillary lesions) and 1 healthy control were collected and approved under the Duke University Health System Institutional Review Board (IRB) protocol Pro00102244 and Pro00079799 as well as exempt due to the nature of otherwise discarded tissue. Skin was manually disrupted using scissors. Tissues were processed using Dispase II (Thermo Fisher Scientific, catalog 17105041, 2.4U/ml) and Collagenase type II (Thermo Fisher Scientific, catalog 17101015, 2 mg/ml) at a 1:1 ratio overnight at 4°C with intermittent vigorous shaking for single-cell isolation. The suspension was filtered through a 70 μm cell strainer. Cells were centrifuged at 1,500 rpm for 12 min at 4°C. Cells were resuspended in 0.4% bovine serum albumin (BSA). Dead cells and erythrocytes were removed using Lympholyte Cell Separation Media, Human (Cedarlane, catalog CL5020) according to the manufacturer's protocol. Cells were centrifuged at 1,500 rpm for 15 min at 4°C and resuspended in 0.4% BSA.

### 10x Single-cell RNA Sequencing

Libraries were prepared following the 10x Genomics Chromium Single Cell 3′ protocols, using either v2 or v3 assays (10X Genomics, catalog 120237, 120236, 100075, 1000153, 120262). Cellometer (Nexcelom - Lawrence, MA) was used to determine the cell viability and concentration to normalize to 1E6 cells/ml. 10,000 cells were targeted for each library. Cells were combined with a master mix that contained reverse transcription reagents. The gel beads carrying the Illumina TruSeq Read 1 sequencing primer, a 16bp 10x barcode, a 12bp unique molecular identifier and a poly-dT primer were loaded onto the chip, together with oil for the emulsion reaction. The Chromium Controller partitioned the cells into nanoliter-scale gel beads in emulsion (GEMS) within which reverse-transcription occurred. All cDNAs within a GEM, i.e., from one cell, shared a common barcode. After the reverse-transcription reaction, the GEMs were broken and the full length cDNAs cleaned with both Silane Dynabeads and SPRI beads (Beckman Coulter, catalog B233180). After purification, the cDNAs were assayed on an Agilent 4200 TapeStation High Sensitivity D5000 ScreenTape (Santa Clara, CA) for qualitative and quantitative analysis. Enzymatic fragmentation and size selection were used to optimize the cDNA amplicon size. Illumina P5 and P7 sequences, a sample index, and TruSeq read 2 primer sequence were added *via* end repair, A-tailing, adaptor ligation, and PCR. The final libraries contained P5 and P7 primers used in Illumina bridge amplification. Sequence was generated using paired end sequencing (one end to generate cell specific, barcoded sequence and the other to generate sequence of the expressed poly-A tailed mRNA) on an Illumina NovaSeq 6000, with a S2 flow cell configured for 28 × 8 × 91 bp reads at a minimum of 50,000 reads/cell.

### Single-cell RNA-sequencing Analysis

The primary analytical pipeline for the scRNA-seq analysis followed the recommended protocols from 10X Genomics. Briefly, we demultiplexed raw base call (BCL) files generated by Illumina sequencers into FASTQ files, upon which alignment to the appropriate reference transcriptome (GRCh38-3.0.0), filtering, barcode counting, and unique molecular identifier counting were performed using 10X Cell Ranger software version 3.1.0. The company protocol uses the Chromium cell barcode to generate feature-barcode matrices encompassing all cells captured in each library. Cell Ranger aggr function was used to pool 3 HS lesion libraries into one combined HS lesion library. The secondary statistical analysis was performed using an R package in Seurat version 3.1.2, which performs quality control and subsequent analyses on the feature-barcode matrices produced by Cell Ranger (44). In Seurat, data was first normalized and scaled after basic filtering for minimum gene and cell observance frequency cut-offs. We then closely examined the data and performed further filtering based on a range of metrics in order to identify and exclude possible multiplets (i.e., instances where more than one cell was present and sequenced in a single emulsified gel bead). The removal of further technical artifacts was performed using regression methods to reduce noise. HS sample reads were integrated for downstream analysis.

After quality control procedures and integration were complete, we performed linear dimensional reduction calculating principal components using the most variably expressed genes in our dataset. Library size and/or the numbers of genes expressed across subsets of cells may necessitate the restriction of cells upon which the variably expressed genes were selected for inclusion when calculating principal components. Significant principal components for downstream analyses were determined through methods mirroring those implemented by Macosko and colleagues, and these principal components were carried forward for two main purposes: to perform cell clustering and to enhance visualization (45). Cells were grouped into an optimal number of clusters for *de novo* cell type discovery using Seurat's FindNeighbors() and FindClusters() functions (44). Graph-based clustering approaches with visualization of cells was achieved through the use of Uniform Manifold Approximation and Projection (UMAP) technique, which reduced the information captured in the selected significant principal components to two dimensions (46).

Differential expression of relevant cell marker genes was visualized on UMAP plot to reveal specific individual cell types. Additional downstream analyses included examining the cellular distribution of a priori genes of interest, closer examination of genes associated with cell clusters, and the refined clustering of cells in order to identify further resolution of cell types, in addition to comparing differences between experiments of different states. Combining multiple libraries using the integration strategy described by Stuart and colleagues allowed for calculation of differential expression, not only between clusters, but within clusters across libraries (47). This allowed for calculation of differential expression within cell types between multiple libraries/samples. Gene expression plots were created using Seurat's VlnPlot(), DotPlot(), FeaturePlot() functions (44, 47).

### Quantitative Polymerase Chain Reaction

HS samples (lesional) and normal skin from HC donors were collected and approved under the Duke University Health System IRB protocol Pro00102244 and Pro00079799 as well as exempt due to the nature of otherwise discarded tissue. RNA was extracted using the Direct-zol RNA Purification Kit (Zymo Research, catalog R2056). cDNA was synthesized using the iScript cDNA Synthesis Kit (Bio-Rad, catalog 1708890). qPCR was performed on 3 HS lesion samples and 3 HC samples in technical duplicates using the Fast SYBR Green Master Mix (Applied Biosystems, catalog 4385617) on a StepOnePlus Real-Time PCR machine. Primers for GBP1, GBP5, IRF1, and STAT1 (Integrated DNA Technologies) are listed in Supplementary Table 2. The melting temperature was 95°C for 3 s and the annealing/extension temperatures were 60°C for 30 s over 40 cycles. Data were normalized to average gene expression of HC skin using the comparative ΔΔ C_T_ method to quantify fold change (48).

Statistical analysis of qPCR results was performed using GraphPad Prism v9.0. Fold change values were log_2_ transformed. Data are represented as mean +/– standard error of the mean of these transformed values. A two-tailed, unpaired samples *t*-test was performed with α = 0.05.

### Immunohistochemistry

HS and normal skin samples were obtained from surgical excisions. The HS skin sample from the buttock was obtained with informed written consent in accordance with the Declaration of Helsinki and with the UT Southwestern IRB protocol STU 072018-067. A normal skin sample was obtained in accordance with the IRB protocol STU 072018-067. The tissues were preserved in paraffin blocks. Samples were deparaffinized in xylene with decreasing ethanol rehydration steps. For epitope retrieval, the slides were immersed in a boiling 10 mm sodium citrate buffer, 0.05% Tween 20, pH 6.0 solution for 30 min. The slides were incubated with a peroxidase suppressor and a blocking buffer from a commercialized IHC kit (Thermo Fisher Scientific, catalog 36000). Samples were then incubated overnight at 4°C with a rabbit monoclonal phospho-STAT1 antibody (Tyr701,clone 58D6) (Cell Signaling Technology, catalog 9167, 1:100), rabbit ISG15 polyclonal antibody (Thermo Fisher Scientific, catalog PA5-31865, 1:100), rabbit polyclonal IFN-β antibody (Novus Biologicals, catalog NBP1-77288, 1:100), rabbit polyclonal OAS2 antibody (Thermo Fisher Scientific, catalog 19279-1-AP, 1:200), or rabbit OASL polyclonal antibody (Thermo Fisher Scientific, catalog PA5-31317, 1:100) at the indicated concentrations. Controls using no primary antibody and rabbit IgG isotype control (Thermo Fisher Scientific, catalog 02-6102) were also completed on the same block of tissue. A goat anti-rabbit IgG secondary antibody (Thermo Fisher Scientific, catalog G-21234) was employed followed by visualization with a DAB/metal concentrate (10x) diluted in a peroxide buffer (Thermo Fisher Scientific, catalog 36000). The samples were counterstained with Vector Nuclear Laboratories VECTOR Nuclear Fast Red (Thermo Fisher Scientific, catalog NC9483816). Images were captured with an Olympus BX46 microscope and CellSens software.

### Immunofluorescence

HS samples (lesional and non-lesional) and normal skin were collected and approved under the Duke University Health System IRB protocol Pro00102244 and Pro00079799 as well as exempt due to the nature of otherwise discarded tissue. Sections were fixed in 4% paraformaldehyde, permeabilized in 0.1% Triton X-100 and blocked in a blocking buffer containing 10% normal goat serum, 5% normal donkey serum, 1% BSA, and 0.05% Triton X-100. Samples were incubated with mouse cytokeratin (KRT)-14 (Novus Biologicals, catalog NBP2-34270, 1:100) and rabbit polyclonal OAS1 (Proteintech, catalog 14955-1-AP, 1:200) or mouse pan-cytokeratin (Abcam, catalog ab226477, 1:250) or unlabeled IgG controls (rabbit IgG, Southern Biotech, catalog 0111-01; mouse IgG, Santa Cruz Biotechnology, catalog sc-3877) overnight at 4°C. Samples were washed with 0.01% TritonX-100 and incubated with AF555-conjugated goat anti-rabbit IgG (Thermo Fisher Scientific, catalog A21428) and AF647-conjugated goat anti-mouse IgG (Thermo Fisher Scientific, catalog A21236) or Cy3-conjugated goat anti-mouse IgG (Thermo Fisher Scientific, catalog A10521) secondary antibodies for 60 min. Samples were then washed and incubated with FITC-conjugated mouse monoclonal CD14 antibody (clone 61D3) (Tonbo Biosciences, catalog 35-0149-T100) or FITC-conjugated isotype IgG control (clone MOPC-21) (Tonbo Biosciences, catalog 35-4714-U100) for 45 min, followed by washing. Nuclear staining was performed with Hoechst solution. Images were captured with an Olympus IX73 inverted microscope and CellSens software.

## Results

### Microarray Dataset Comparison Between HS Lesions and Chronic Non-Healing Wounds Reveals Shared Enrichment in Genes Involving Innate Immunity and Phagocytosis

**Figure 1:**
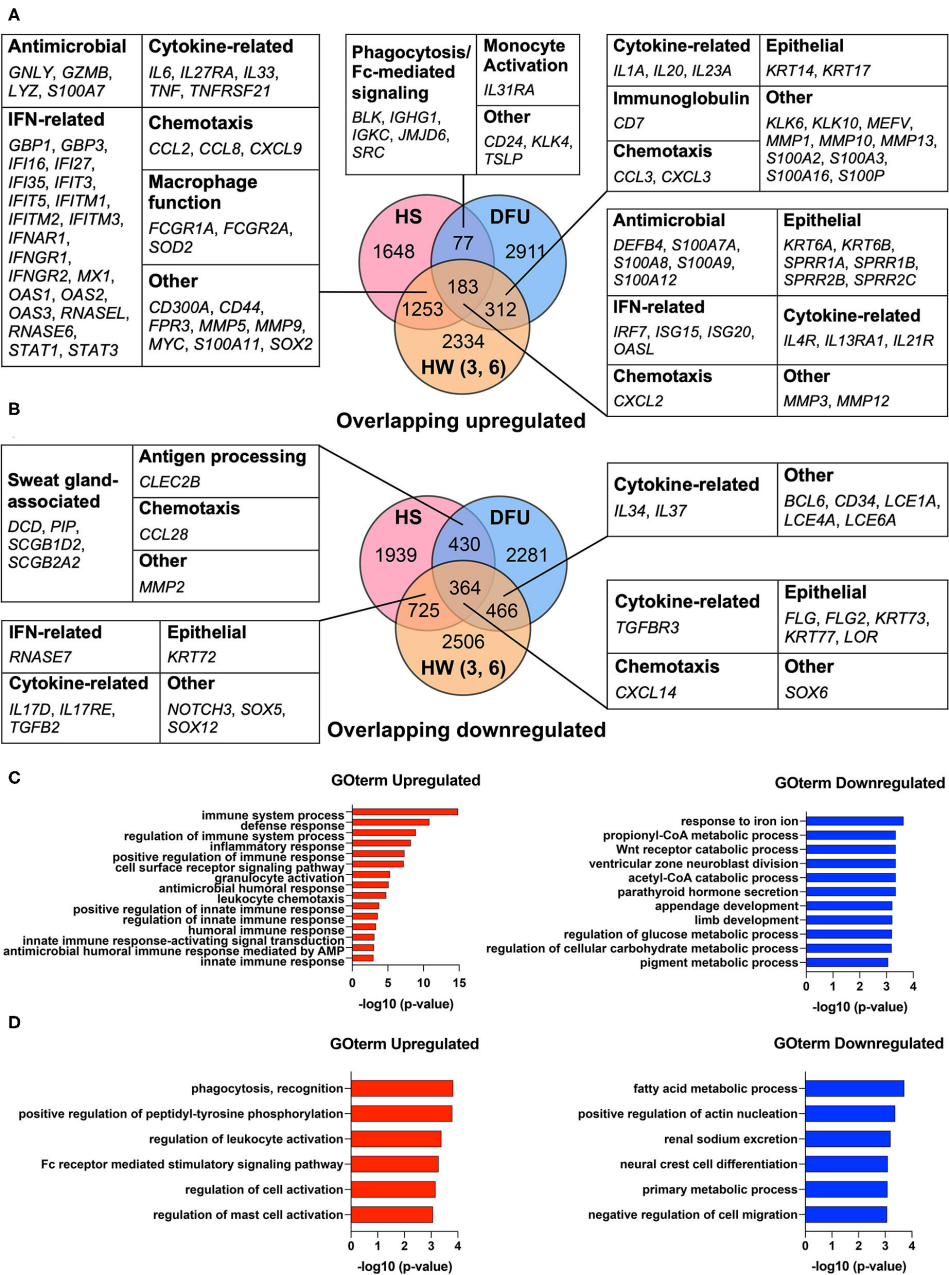
HS and DFU share an increased innate immune response enriched in macrophage function. **(A)** Notable upregulated DEGs shared between HS, HW, and DFU organized by biological function. Numbers in overlapping circles indicate the amount of shared upregulated genes for that respective comparison. Comparisons involving the HW dataset included “day 3 vs. day 1”, “day 6 vs. day 1”, or “day 3 and 6 vs. day 1”. **(B)** Notable downregulated DEGs shared between HS, HW, and DFU organized by biological function. **(C)** Enriched biological processes in the analysis of HS, HW, and DFU. DEGs in the HS dataset were used as the background dataset. **(D)** Enriched biological processes in the analysis of HS and DFU only. DEGs in the HS dataset were used as the background dataset. DEG, differentially expressed gene; HW (3, 6), healing wound, post-wound days 3 and/or 6 vs. day 1; DFU, diabetic foot ulcer.

Our previous work found that HS shares an antimicrobial and SG gene signature with skin wounds that go on to heal (16). While this transcriptomic analysis showed that interferon stimulated genes (ISGs) were elevated in HS lesions as well as in the inflammatory phase of healing wounded skin, we did not address how such an immune response may relate to a chronic wound environment (16). Therefore, we here included an additional dataset from DFUs (GSE80178) (31) in the comparison between HS lesions (GSE72702) (30) and HW in the inflammatory phase (GSE97615) (32) to characterize the shared characteristics of HS with healing and non-healing skin. During wound healing, the inflammatory phase is important for controlling microbial colonization and eliminating cellular and microbial debris *via* phagocytosis (14). Analysis of the DFU dataset identified DEGs with an adjusted *p*-value ≤0.05 in the comparisons between both “Diabetic Foot Ulcer vs. Diabetic Foot Skin” and “Diabetic Foot Ulcer vs. Foot Skin,” but not in “Diabetic Foot Skin vs. Foot Skin” (31). For the HS dataset, we identified DEGs between HS lesions and non-lesional HS skin with an adjusted *p*-value ≤0.05 (30). Finally, in the HW dataset, either day 3 or 6 post-wound skin samples were compared with unwounded “day 1” samples to determine DEGs with an adjusted *p*-value ≤0.05 (32). We found that many DEGs involved in IFN pathways were upregulated among all three datasets, including *IRF7, ISG15, ISG20*, and *OASL* (Figure 1A). Similarly, many DEGs that encode for AMPs were upregulated among these datasets, such as *DEFB4, S100A7A, S100A8*, and *S100A9* (Figure 1A). In the overlap analysis between HS and DFU alone, we determined that DEGs associated with phagocytosis, Fc-mediated signaling, and monocyte activation were upregulated (Figure 1A). However, DEGs associated with SG function, including *DCD* and *PIP*, were downregulated in this comparison (Figure 1B). Together, our data indicate that HS and DFU share a gene signature of antimicrobial activity, IFN signaling, and macrophage function with diminished expression of SG associated genes.

To better understand the biological processes that are significantly altered in HS, DFU, and the inflammatory phase of HW, we performed Gene Ontology Enrichment Analysis (GOrilla) among DEGs upregulated across all three datasets using DEGs in the HS dataset as a background set (49). The same was performed for downregulated DEGs across all three datasets (49). We found that innate immunity-related GO terms, including “positive regulation of innate immune response” (GO:0045089, *p* = 1.53E-5), “regulation of innate immune response” (GO:0045088, *p* = 2.43E-5), and “innate immune response” (GO:0045087, *p* = 7.05E-5), were significantly enriched (Figure 1C). GO terms relating to cellular metabolic processes were among the most significantly reduced in the shared downregulated DEGs (Figure 1C). To focus our analysis on the shared characteristics between HS and chronic wounds, we used GOrilla to compare shared upregulated DEGs in the HS and DFU datasets only. Among the most significantly enriched processes included “phagocytosis, recognition” (GO:0006910, *p* = 1.47E-4) and “Fc receptor mediated stimulatory signaling pathway” (GO:0002431, *p* = 5.16E-4) (Figure 1D). Overall, our results suggest that the innate immune processes observed in HS and DFUs are enriched in pathways related to phagocytosis and Fc receptor-mediated signaling.

### Single-cell RNA Sequencing Uncovers Upregulation of Immune System Processes in Immune and Skin-structural Cells

**Figure 2:**
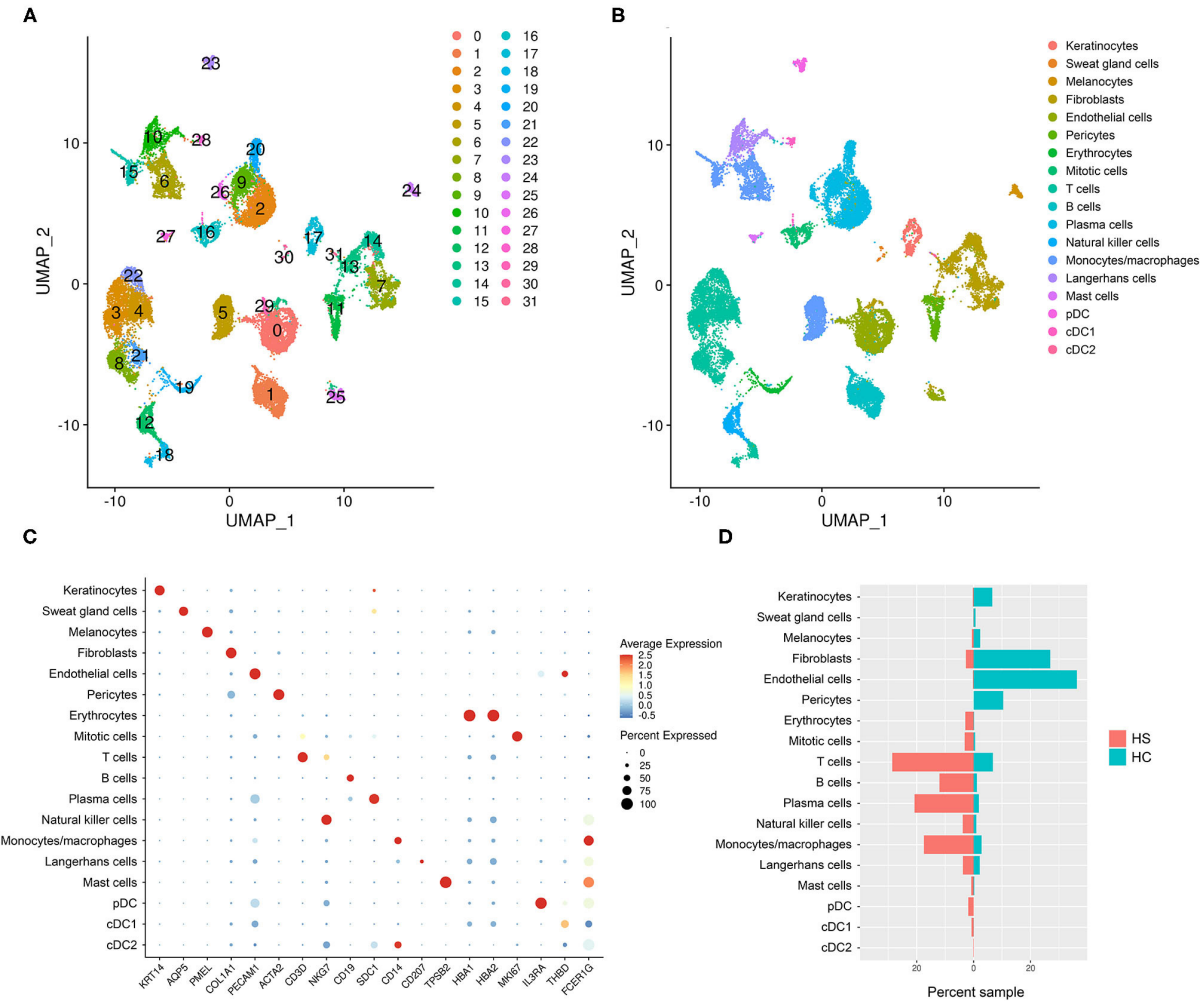
scRNA-sequencing reveals a relative abundance of immune cells in HS skin lesions. **(A)** Unsupervised clustering represented in a UMAP plot of skin samples collected from 3 patients with HS and 1 HC. **(B)** UMAP plot of clusters grouped by cellular identity. **(C)** Dot plot of the percent expressed and average expression of signature genes for clusters grouped by cellular identity. **(D)** Sample distribution of cell populations by percent of total sample organized by whether they came from HS or HC skin. cDC, conventional dendritic cell; HC, healthy control; pDC, plasmacytoid dendritic cell. “Mitotic cells” were defined as KI67-expressing cells in the mitotic phase of the cell cycle.

To examine the cell types and cell-specific transcriptome of HS, we performed scRNA-seq on cells isolated from axillary lesions excised from 3 patients with severe HS and normal skin from 1 healthy control (HC). A total of 23,911 cells in HS lesions and 9,713 cells in HC skin were sequenced, with a median of 1,842 genes detected in HS and 1,504 genes detected in HC. We performed unsupervised clustering analysis which grouped cells into 32 clusters (Figure 2A). From these clusters, we identified 18 cell types using signature genes (Figures 2B,C, and Supplementary Table 3). The majority of cells in the control samples were epithelial cells (keratinocytes, melanocytes, and SG cells), fibroblasts, and the dermal components of blood vessels, including endothelial cells and pericytes (Figure 2D and Supplementary Figure 1). On the other hand, immune cells comprised a greater proportion of HS samples than of control (Figure 2D and Supplementary Figure 1). GOrilla analysis on DEGs in HS relative to HC for each cell type revealed significantly enriched biological processes; the top 15 GO terms for select epithelial and immune cells are represented in Supplementary Figure 2. Notably, “immune effector process” (GO:0002252) and “immune response” (GO:0006955) were present not only in immune cells clusters such as monocytes/macrophages, dendritic cells (DCs), Langerhans cells (LCs), and B/T cells, but also in keratinocytes and SG cells (Supplementary Figure 2). Humoral and complement-related GO terms were also enriched in the cell types represented in Supplementary Figure 2. Biological processes related to phagocytosis, including “phagocytosis, recognition” (GO:0006910) and “phagocytosis, engulfment” (GO:0006911) were among the top 15 most enriched GO terms in all cell types represented in Supplementary Figure 2.

Gudjonsson and colleagues have recently reported enrichment of humoral and complement-related processes in HS skin and performed in-depth analysis of these immune compartments at a single-cell level (23). Likewise, although T cells were one of the greatest contributors to the immune cell population in our scRNA-seq analysis, their transcriptomic profile has been well-described by Lowe and colleagues (Figure 2D) (50). Given that our dataset comparison between HS lesions, DFUs, and HWs in the inflammatory phase revealed a shared antiviral, antimicrobial, and phagocytic gene signature, we next focused our attention on monocytes/macrophages and other pertinent innate immune components in our scRNA-seq analysis.

### Activated M1 Macrophages Enriched in Genes Relating to Phagocytosis, Respiratory Burst, and Antibody-Dependent Cellular Cytotoxicity Are Present in HS Skin Lesions

A key feature in the dataset comparison between HS and HW in the inflammatory phase and between HS and DFU was the shared upregulation of DEGs related to macrophage function, phagocytosis, and Fc receptor-mediated signaling (Figure 1A). Monocytes/macrophages not only contributed substantially to the cellular composition of HS lesions in our scRNA-seq analysis, but also play critical roles in wound healing and skin inflammation (Figure 2D). Therefore, we examined this cell population in depth through single-cell profiling.

**Figure 3:**
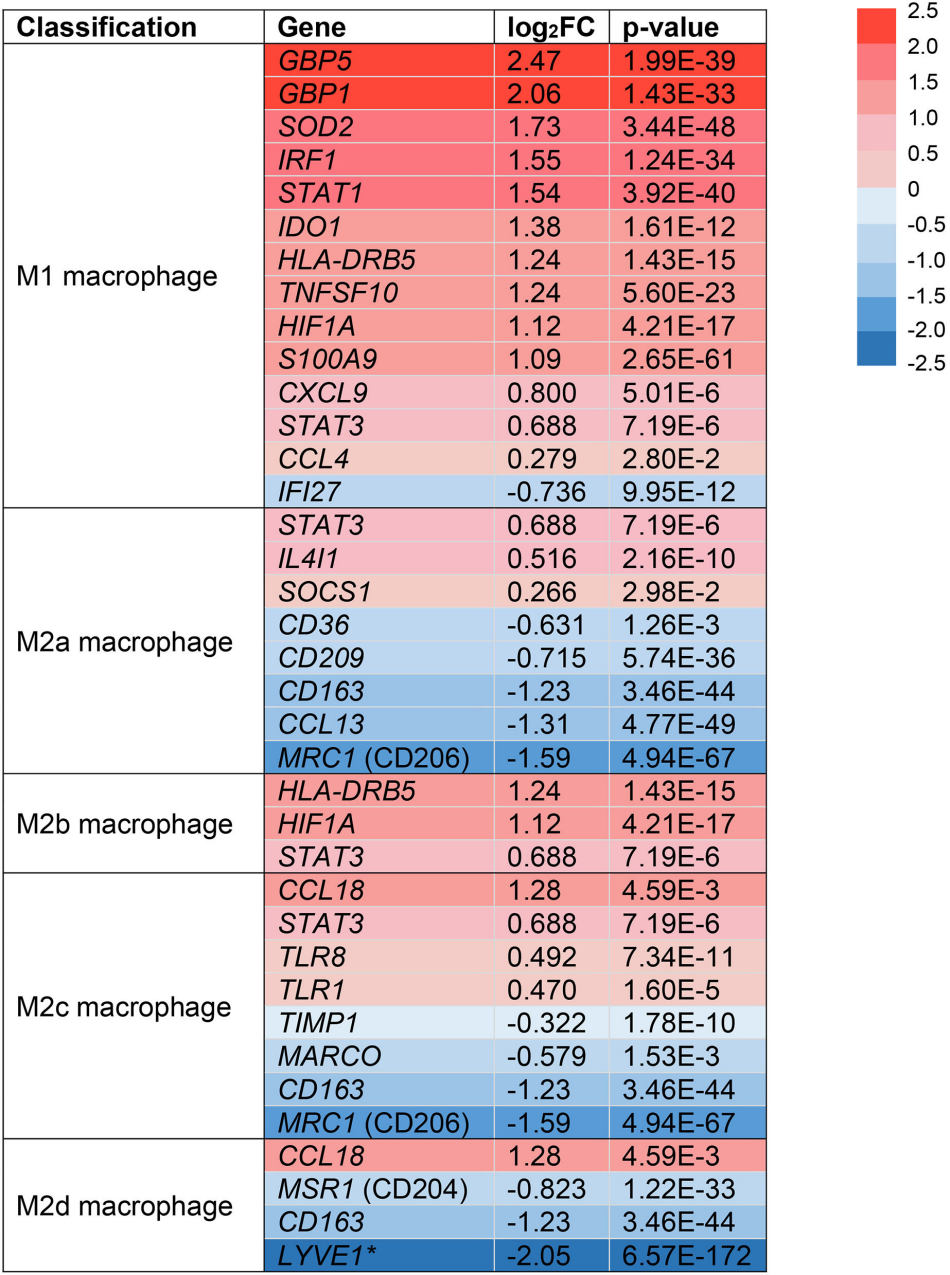
Monocyte/macrophage populations are transcriptionally polarized toward an M1 macrophage phenotype in HS skin. log_2_FC of gene expression was calculated by comparing HS samples to HC skin within the monocyte/macrophage clusters. Values that depict an increased log_2_FC gene expression in HS relative to HC are colored in red and those that depict a decreased expression are colored in blue. *Expressed in tumor-associated macrophages. FC, fold change; HC, healthy control.

Following extravasation from the blood, monocytes can differentiate into macrophages in tissues like the skin and under the influence of micro-environmental signals that drive the acquisition of a functional phenotype (51). In the skin, macrophages are important resident immune cells that can be classified by a pro-inflammatory (M1) or anti-inflammatory (M2) phenotype (12). Due to their dichotomous functions across multiple stages of wound healing, we sought to identify if monocytes/macrophages in HS lesions are polarized toward an M1 or M2 phenotype (14). We analyzed the expression of surface markers and transcription factors in the monocyte/macrophage clusters from our scRNA-seq analysis (Figure 3). Multiple genes associated with M1-like macrophages were upregulated in HS lesions as shown in Figure 3 (11). Notably, we found upregulation of MHC class II (*HLA-DRB5*, log_2_FC = 1.24, *p* = 1.43E-15) and *STAT1* (log_2_FC = 1.54, *p* = 3.92E-40) genes, which are characteristic of M1 macrophages (Figure 3) (11). In agreement with M1 macrophage polarization in HS skin, genes characteristic of a M2 phenotype were downregulated (Figure 3) (11). For instance, we observed decreased expression of genes encoding for the M2 macrophage receptor *CD163* (log_2_FC = −1.23, *p* = 3.46E-44) and Mannose Receptor C-Type 1 *MRC1* (log_2_FC = −1.59, p = 4.59E-67) (Figure 3) (11). These genes are important for the anti-inflammatory response *via* scavenging of hemoglobin-haptoglobin complexes and endocytosis of pro-inflammatory glycoproteins, respectively (52, 53).

**Figure 4:**
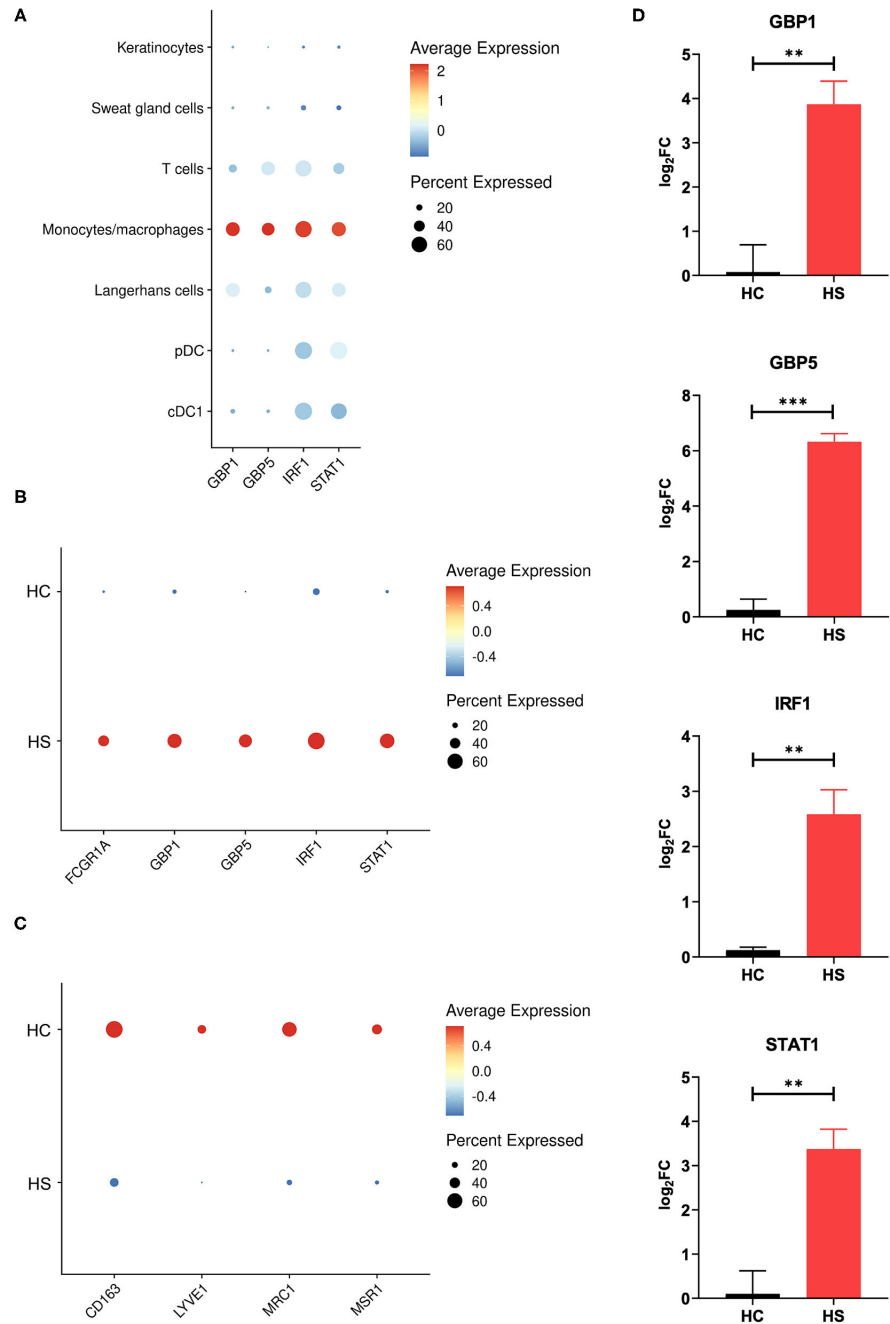
M1 macrophage genes are more abundantly expressed in HS skin. **(A)** Dot plot showing the percent expressed and average expression of select M1 macrophage genes between key cellular populations in HS skin. **(B)** Dot plot showing the percent expressed and average expression of select M1 macrophage genes between HS and HC skin in monocyte/macrophage populations. **(C)** Dot plot showing the percent expressed and average expression of select M2 macrophage genes between HS and HC skin in monocyte/macrophage populations. **(D)** qPCR of *GBP1* (log_2_FC = 3.87, **p < 0.01), *GBP5* (log_2_FC = 6.33, ***p < 0.005), *IRF1* (log_2_FC = 2.58, **p < 0.01), and *STAT1* (log_2_FC = 3.38, **p < 0.01) in HS lesions and HC skin. log_2_FC is expressed as an average of skin samples from 3 HS or HC patients relative to HC skin. Measurements were collected in duplicate. Data is represented as the mean log_2_FC value +/– standard error of the mean. cDC, conventional dendritic cell; HC, healthy control; pDC, plasmacytoid dendritic cell.

Between cell populations, key M1 macrophage genes such as *GBP5* and *STAT1* had high average expression and percentage expression in monocytes/macrophages but not in other immune cells (Figure 4A). To further investigate the apparent profile of M1 transcriptional polarization, we analyzed the average expression level and proportion of monocytes/macrophages expressing M1 and M2 macrophage genes between HS and HC skin. Indeed, M1 macrophage genes appeared to be more highly expressed across a greater proportion of cells in HS compared to HC skin (Figure 4B). On the contrary, there was reduced and less abundant expression of M2 macrophage genes in HS compared to HC skin (Figure 4C). At a bulk transcriptional level, genes associated with M1-like macrophages such as *GBP5* and *STAT1* were upregulated in HS tissue (Figure 4D). Given that these genes were most strongly and abundantly expressed by monocytes/macrophages compared to other cellular populations, the upregulation of M1 macrophage genes in HS lesions likely indicates transcriptional polarization of macrophages.

**Figure 5:**
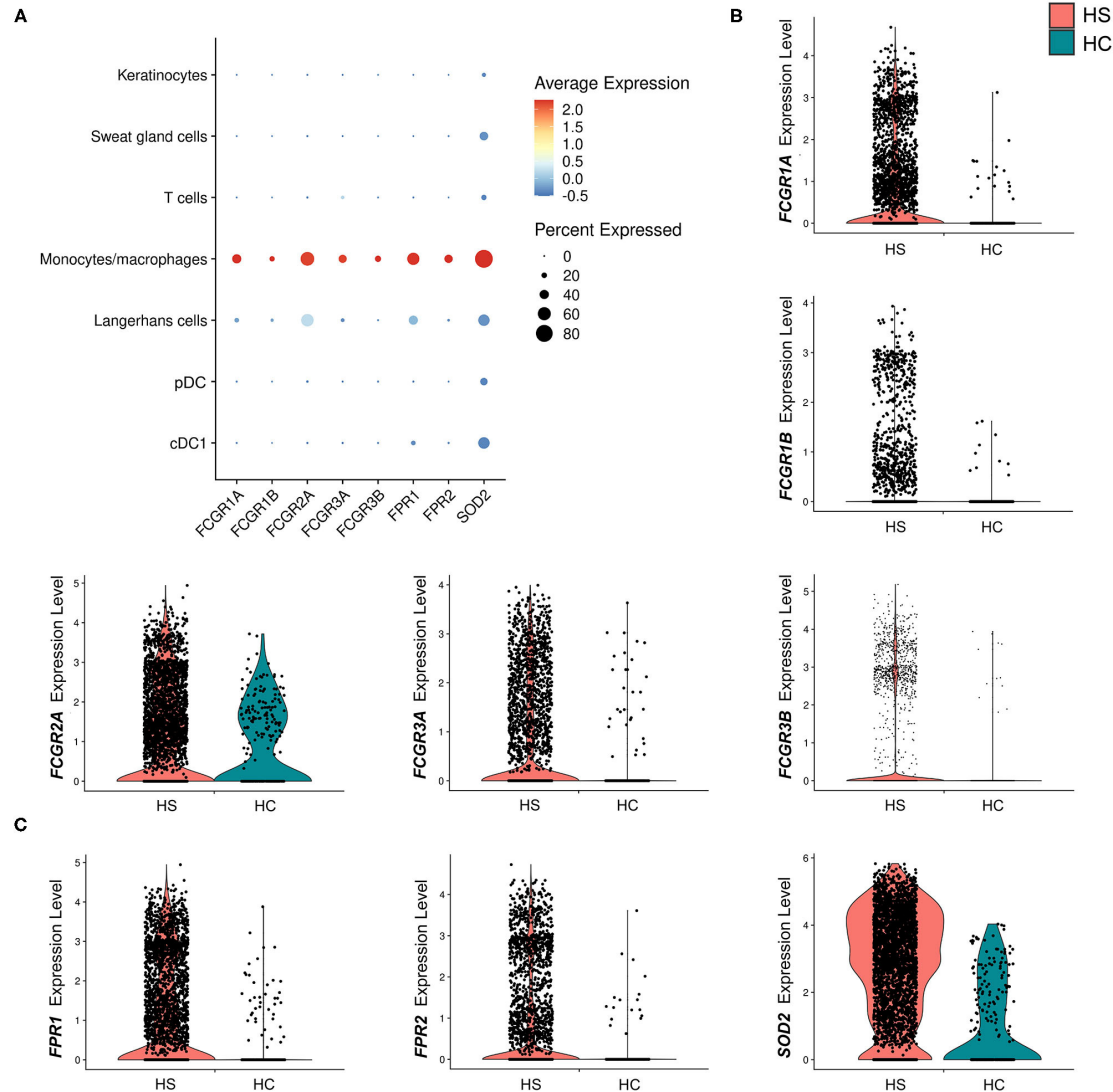
Macrophages in HS lesions show upregulation of markers associated with Fc signaling and metabolic activity. **(A)** Dot plot showing the percent expressed and average expression of genes related to phagocytosis and metabolic activity between key cellular populations in HS skin. **(B)** Violin plots for the monocyte/macrophage cluster of genes related to Fc signaling. **(C)** Violin plots for the monocyte/macrophage cluster of genes related to respiratory burst. cDC, conventional dendritic cell; HC, healthy control; pDC, plasmacytoid dendritic cell.

Many key functions of monocytes/macrophages are mediated *via* the Fc signaling pathway. Ligand binding to Fc receptor (FcR) leads to a signaling cascade mediated by immunoreceptor tyrosine-based motifs, resulting in activation or inhibition of an immune response (54). We found that receptor genes associated with inflammatory immune responses *via* the FcγR signaling pathway were upregulated in the monocyte/macrophage population within HS skin. These genes included *FCGR1A* (log_2_FC = 1.28, *p* = 5.09E-24), *FCGR1B* (log_2_FC = 0.732, *p* = 1.97E-9), *FCGR2A* (log_2_FC = 0.548, *p* = 4.00E-4), *FCGR3A* (log_2_FC = 0.704, *p* = 1.11E-11), and. *FCGR3B* (log_2_FC = 1.23, *p* = 3.26E-12) (Figures 5A,B and Table 1). Genes involving the signaling cascade were also upregulated, including *LYN* (log_2_FC = 1.24, *p* = 2.33E-32), *PTPN6* (log_2_FC = 0.708, *p* = 1.62E-10), and *SYK* (log_2_FC = 0.266, *p* = 2.79E-3) (Table 1) (54).

**Table 1:** Genes related to Fcγ signaling are upregulated in monocytes/macrophages of HS lesions compared to healthy control skin.

**Gene**	**Protein**	**log_**2**_FC**	***p*-value**	**Function in Fc signaling**	**Downstream effects**	**Ref**.
*FCGR1A*	FcγRIa (CD64a)	1.28	5.09E-24	Activating receptor	ADCC; phagocytosis; cytokine secretion	(54–56)
*FCGR1B*	FcγRIb (CD64b)	0.732	1.97E-9	Activating receptor	ADCC; phagocytosis; cytokine secretion	(54–56)
*FCGR2A*	FcγRIIa (CD32a)	0.548	4.00E-4	Activating/inhibitory receptor	Phagocytosis; cytokine secretion	(56, 57)
*FCGR3A*	FcγRIIIa (CD16a)	0.704	1.11E-11	Activating/inhibitory receptor	ADCC; phagocytosis; cytokine secretion; respiratory burst	(58–60)
*FCGR3B*	FcγRIIIb (CD16b)	1.23	3.26E-12	Activating/inhibitory receptor	Phagocytosis; neutrophil degranulation; respiratory burst	(59, 60)
*LYN*	Lyn	1.24	2.33E-32	Tyrosine kinase	Proliferation; migration; cytokine secretion (activation/inhibition)	(61)
*PTPN6*	SHP-1	0.708	1.62E-10	Tyrosine phosphatase	Phagocytosis (inhibition)	(62)
*SYK*	SYK	0.266	2.79E-3	Tyrosine kinase	Cytokine secretion; respiratory burst (activation)	(63)

*For genes encoding Fcγ receptors, activation/inhibition function is represented in “Function in Fc signaling” column. For genes encoding tyrosine kinase/phosphatase, activation/inhibition function is represented in “Downstream effects” column. log_2_FC of gene expression was calculated by comparing HS samples to healthy control skin within the monocyte/macrophage clusters. ADCC, antibody-dependent cellular cytotoxicity; FC, fold change; SHP-1, Src homology region 2 domain-containing phosphatase-1*.

Activation of macrophages by FcγR signaling leads to phagocytosis, oxidative burst, cytokine/chemokine production, and ADCC (54). Phagocytosis is a common process in wound healing that eliminates apoptotic myofibroblasts and vascular cells to maintain tissue homeostasis (64). It is known that macrophages promote wound healing through efferocytosis, a process that involves the engulfment of apoptotic cells *via* phagocytosis (65). Dysfunction of this process leads to accumulation of apoptotic cells and prolongs the inflammatory phase of wound healing (65). Additionally, impaired efferocytosis is associated with increased expression of pro-inflammatory cytokines and decreased expression of anti-inflammatory cytokines (65). The importance of this function led us to examine genes relating to phagocytosis in macrophages within HS lesions (Figures 5A,B). We found upregulation of the *FCGR1A* (log_2_FC = 1.28, *p* = 5.09E-24) and *FCGR1B* (log_2_FC = 0.732, *p* = 1.97E-9) genes, which encode for FcγRI (CD64), a receptor upregulated on M1 macrophages in chronic inflammatory diseases (Figures 5A,B, and Table 1) (55). Impaired efferocytosis prevents M2 macrophages from producing anti-inflammatory cytokines such as IL-10 (not differentially expressed in HS sample), which is required for normal wound healing (66). As expected, genes associated with M2 macrophage phagocytosis of apoptotic cells were downregulated in the HS sample, including *CD163* (log_2_FC = −1.23, *p* = 3.46E-44) and *CD209* (log_2_FC = −0.715, *p* = 5.74E-36) (Figure 3) (52, 67). The transcriptional profile of increased phagocytosis in M1-like macrophages but decreased phagocytosis in M2-like macrophages may contribute to continuous inflammation and impaired wound healing in longstanding HS skin.

We found that genes involved in the generation of reactive oxygen species (ROS) *via* N-formyl-Met-Leu-Phe were upregulated (*FPR1*, log_2_FC = 1.38, *p* = 6.32E-30; *FPR2*, log_2_FC = 1.36, *p* = 7.66E-19) (Figure 5C). In addition, monocyte/macrophage expression of superoxide dismutase (SOD) 2 (log_2_FC = 1.73, *p* = 3.44E-48) was greater in HS than in HC skin (Figure 5C). In respiratory burst, the enzyme SOD converts superoxide radicals into hydrogen peroxide, which has antimicrobial activity.

In addition to direct antimicrobial activity *via* phagocytosis and respiratory burst, monocytes and macrophages can mediate antimicrobial and antitumor immunity *via* ADCC. *In vitro*, ADCC in monocytes is FcγRIII (CD16)-dependent and involves β2-integrin, which is encoded by *ITGB2* (58). This gene was upregulated (log_2_FC = 0.792, *p* = 8.01E-17) in the monocyte/macrophage cluster within HS lesions. M1 macrophages can prime NK cells and T cells to increase their cytotoxicity *via* ADCC (68, 69). *GZMK* (log_2_FC = 2.24, *p* = 1.42E-26) was the third most upregulated gene in NK cells. *GZMA* (log_2_FC = 0.514, *p* = 8.38E-5) was slightly upregulated in NK cells. Granzymes (*GZMA*, log_2_FC = 0.817, *p* = 2.21E-42; *GZMB*, log_2_FC = 0.458, *p* = 7.45E-9; *GZMK*, log_2_FC = 1.28, *p* = 4.89E-24; *GZMH*, log_2_FC = 0.575, *p* = 6.38E-12) and perforin (*PRF1*, log_2_FC = 0.383, *p* = 3.66E-16) were transcriptionally increased in T cell populations within HS lesions. Overall, our data show that the monocytes/macrophages within HS lesions polarize toward an M1 phenotype and have increased transcription of genes involved in pro-inflammatory and antimicrobial functions, such as phagocytosis, respiratory burst, and activation of ADCC.

### Key Cell Populations in HS Lesions Demonstrate Transcriptional Changes Associated With a Type 1 Immune Response

The transcriptomic signature of M1 macrophage polarization and NK cell and T cell activity may suggest a skew toward a type 1 immune response in HS samples. Type 1 immunity is important for protection against intracellular microbes *via* activation of NK cells, Th1 cells, and mononuclear phagocytes (70). We identified upregulation of the chemokine gene *CXCL9* (log_2_FC = 0.800, *p* = 5.01E-6) in the monocyte/macrophage population (Figure 3). These chemokines are stimulated by IFNs and function in the recruitment of natural killer (NK) cells and effector T cells *via* interaction with the CXCR3 receptor, whose gene was upregulated in the NK cell cluster (log_2_FC = 0.773, p = 1.22E-11) and T cell cluster (log_2_FC = 0.414, *p* = 4.93E-32) (71). We also observed upregulation of *CXCR6* (log_2_FC = 1.24, *p* = 3.35E-83), *IRF1* (log_2_FC = 0.610, *p* = 3.13E-65) and *STAT1* (log_2_FC = 0.568, *p* = 3.14E-52) in T cells within HS lesions. These genes are involved in Th1 differentiation, response to IFN-γ, and homing to inflamed tissue (70, 72, 73).

We further investigated the transcriptional changes associated with a type 1 immune response in LC, a unique population of tissue-resident immune cells that are ontogenically linked to macrophages (74). CD1a+ LC have been found at significantly increased levels in chronic HS lesions compared to early lesions (75). Within the LC population, we found increased expression of the chemokine genes *CXCL9* (log_2_FC = 1.45, *p* = 4.25E-12) and *CXCL10* (log_2_FC = 0.661, *p* = 2.04E-6) (Supplementary Table 4). These chemokines are induced by IFN-γ to recruit immune cells to sites of inflammation and regulate differentiation of naive T cells to a Th1 phenotype (71). We also identified upregulation of *STAT1* (log_2_FC = 0.995, *p* = 1.6E-33) and *IL18* (log_2_FC = 0.541, *p* = 6.62E-13) in LCs; the latter encodes for a pro-inflammatory cytokine that promotes type 1 immune responses *via* induction of IFN-γ production by Th1 cells in the presence of IL-12 (Supplementary Table 4) (76).

Importantly, upregulation of type 1 immune response genes in HS lesions does not preclude the expression of genes associated with other types of immune responses. For instance, *CCL18* (log_2_FC = 1.28, *p* = 4.59E-3) was strongly expressed in monocytes/macrophages and is associated with type 2 immune responses involving Th2 cells that produce IL-4, IL-5, and IL-13 (77). Transcriptional and cellular changes associated with the immune responses involving Th2 and Th17 cells in HS is outside the scope of this paper but should be investigated in future studies.

### Type I and Type II Interferon-Stimulated Genes Are Upregulated in Monocytes/Macrophages Within HS Lesions

In our comparative analysis of the HS, DFU, and HW datasets, we identified the shared upregulation of many DEGs related to IFN signaling, including IFN-γ receptor genes, ISGs, and signal transducer and activator of transcription (STAT) genes (Figure 1A). IFNs are potent regulators of cutaneous immune responses to viral infection. Binding of IFN to receptor activates STAT complexes and other signaling cascades, which regulate transcription of ISGs. A previous scRNA-seq analysis by Gudjonsson and colleagues demonstrated a prominent type II IFN (IFN-γ) signature in HS skin, notably in T cells and keratinocytes (23). Given that IFN-γ can induce activation, FcγR expression, and polarization toward an M1 phenotype in macrophages, we sought to further characterize the downstream effects of IFN activation in this cell population (78). To do so, we first examined ISGs with γ interferon activation sites (GAS), which are critical for transcription of genes responding to type II IFN signaling, within their promoter regions (Table 2) (79). We found that many ISGs with GAS were indeed upregulated in the monocyte/macrophage population within HS lesions (Table 2). Many of these ISGs also possessed interferon-sensitive response elements (ISREs) within their promoter regions, which stimulate transcription of genes related to type I IFN signaling, the two most notable of which are IFN-α and IFN-β (Table 3) (79). Importantly, we found that certain upregulated ISGs in our analysis of monocytes/macrophages only possessed ISRE or GAS sites within their promoter regions, while others harbored both GAS and ISREs (Tables 2, 3). Together, our data demonstrate that HS skin lesions have increased expression of ISGs induced by both type I and type II IFNs in the monocyte/macrophage population.

**Table 2:** Interferon-γ stimulated genes are upregulated in monocytes/macrophages from HS lesions compared to healthy skin.

**Gene**	**log_**2**_FC**	***p*-value**
*GBP1*	2.06	1.43E-33
GBP2^*^	1.03	7.88E-15
*GBP3*	0.466	2.40E-6
*GBP4*	1.47	1.23E-25
*GBP5*	2.47	1.99E-39
*B2M*	0.888	2.66E-74
HLA-A^*^	0.914	1.75E-51
HLA-B^*^	0.985	2.07E-70
HLA-C^*^	1.60	1.95E-83
HLA-E^*^	0.440	1.73E-9
HLA-F^*^	0.905	1.69E-20
*HLA-DRB5*	1.24	1.43E-15
*IFI30*	0.288	2.72E-5
IRF1^*^	1.55	1.24E-34
IRF2^*^	0.475	2.31E-5
IRF7^*^	0.518	2.70E-8
IRF9^*^	0.543	1.46E-6
*FCGR1A*	1.28	5.09E-24
*FCGR1B*	0.732	1.97E-9
OAS1^*^	0.304	3.47E-7
*SP100*	0.578	2.58E-6
*ICAM1*	0.355	2.49E-3
*CD44*	0.722	2.09E-13
*PTAFR*	0.676	2.71E-10
*PML*	0.381	1.92E-4
*TRIM21*	0.305	2.47E-5
*TRIM22*	0.459	1.17E-2
*TRIM25*	0.312	1.46E-2
*TRIM38*	0.599	3.20E-9

*Upregulated genes have γ interferon-activated sequence (GAS) elements in the promoter regions of IFN-stimulated genes (ISGs). log_2_FC of gene expression was calculated by comparing HS samples to healthy control skin within the monocyte/macrophage clusters*.

* *These genes have both interferon-sensitive response elements (ISREs) and GAS in their promoters*.

**Table 3:** Type I IFN-stimulated genes are upregulated in monocytes/macrophages from HS lesions compared to healthy control skin.

**Gene**	**log_**2**_FC**	***p*-value**
GBP2^*^	1.03	7.88E-15
HLA-A^*^	0.914	1.75E-51
HLA-B^*^	0.985	2.07E-70
HLA-C^*^	1.60	1.95E-83
HLA-E^*^	0.440	1.73E-9
HLA-F^*^	0.905	1.69E-20
*IFIT2*	1.32	2.36E-11
*IFIT3*	1.28	5.09E-15
*IFIT5*	0.299	1.84E-3
*IFITM2*	0.835	6.38E-4
*IFI6*	0.577	7.82E-9
*IFI35*	0.405	5.26E-5
IRF1^*^	1.55	1.24E-34
IRF2^*^	0.475	2.31E-5
IRF7^*^	0.518	2.70E-8
IRF9^*^	0.543	1.46E-6
*ADAR*	0.990	1.60E-15
*PSMB8*	0.877	1.50E-16
*XAF1*	0.724	1.27E-7
*ISG15*	0.403	2.72E-3
*ISG20*	0.968	3.51E-7
*MX1*	0.278	1.06E-5
*MX2*	0.519	3.02E-4
OAS1^*^	0.304	3.47E-7

*Upregulated genes have interferon-sensitive response elements (ISREs) in the promoter regions of IFN-stimulated genes (ISGs). log_2_FC of gene expression was calculated by comparing HS samples to healthy control skin within the monocyte/macrophage clusters*.

* *These genes have both ISREs and γ-activated sequences (GAS) in their promoters*.

Though previous studies have uncovered a type I IFN signature in HS skin, the source of these IFNs is unclear (23, 25). An abundant source of type I IFNs are DCs, specifically the pDC subtype (80). With this important function in mind, we investigated the 32 initially identified clusters and defined 3 of these clusters as the DC population (Figures 2A,B). Notably, in the U-MAP plot, these 3 clusters were considerably apart, therefore, we kept these clusters separate in our analysis of different cell populations (Figures 2A,B). To further explore these differences, we found signature genes of different subsets of DCs using differentially-expressed markers (Supplementary Table 3) (81). Cluster 23 showed high levels of *CD4, CLEC4C* (CD303), *FCER1G* (FcεRI), *IL3R* (CD123), *NRP1, and TNFRSF21*, compatible with a well-characterized pDC phenotype (Supplementary Table 3) (81). “Classical” or “conventional” DCs (cDC) can be classified in two types: cDC1 and cDC2 (81). cDC1-like cells in cluster 28 expressed *BTLA, CADM1, CLEC9A*, and *THBD* (Supplementary Table 3). Finally, cluster 31 expressed genes associated with a cDC2-like phenotype, such as *CD68, CLEC4A, CLEC7A, FCER1G* (FcεRI), and *SIRPA* (Supplementary Table 3). Notably, we observed the presence of only 1 pDC in the HC sample while the HS sample contained hundreds of pDCs (Supplementary Figure 1A). In addition, cDC2 cells were absent in the HC sample, therefore, differential gene expression in HS vs. HC skin was unable to be performed for this cell population (Supplementary Figure 1A). Given that pDCs are typically infrequent or absent in normal skin, the presence of this subtype may contribute to the type I IFN gene signature found within HS lesions (82).

### Antiviral Proteins and Antimicrobial Peptides and Proteins Are Transcriptionally Upregulated in Distinct Cell Populations Within HS Lesions

As previously described, comparison between the HS, HW, and DFU datasets revealed a prominent IFN-related gene signature (Figure 1A). Canonically, IFN signaling in the skin results in antiviral activity *via* production of AVPs by keratinocytes and skin immune cells (83). AVP production may also be stimulated in an IFN-independent manner (83). For instance, our group demonstrated that AVPs in the epidermis can be induced in keratinocytes in response to IFNs and the IFN-independent cytokine IL-27 (84). Given the shared upregulation of antiviral DEGs across the HS, HW, and DFU datasets, we sought to characterize AVP expression in multiple cell populations at a single-cell resolution.

**Figure 6:**
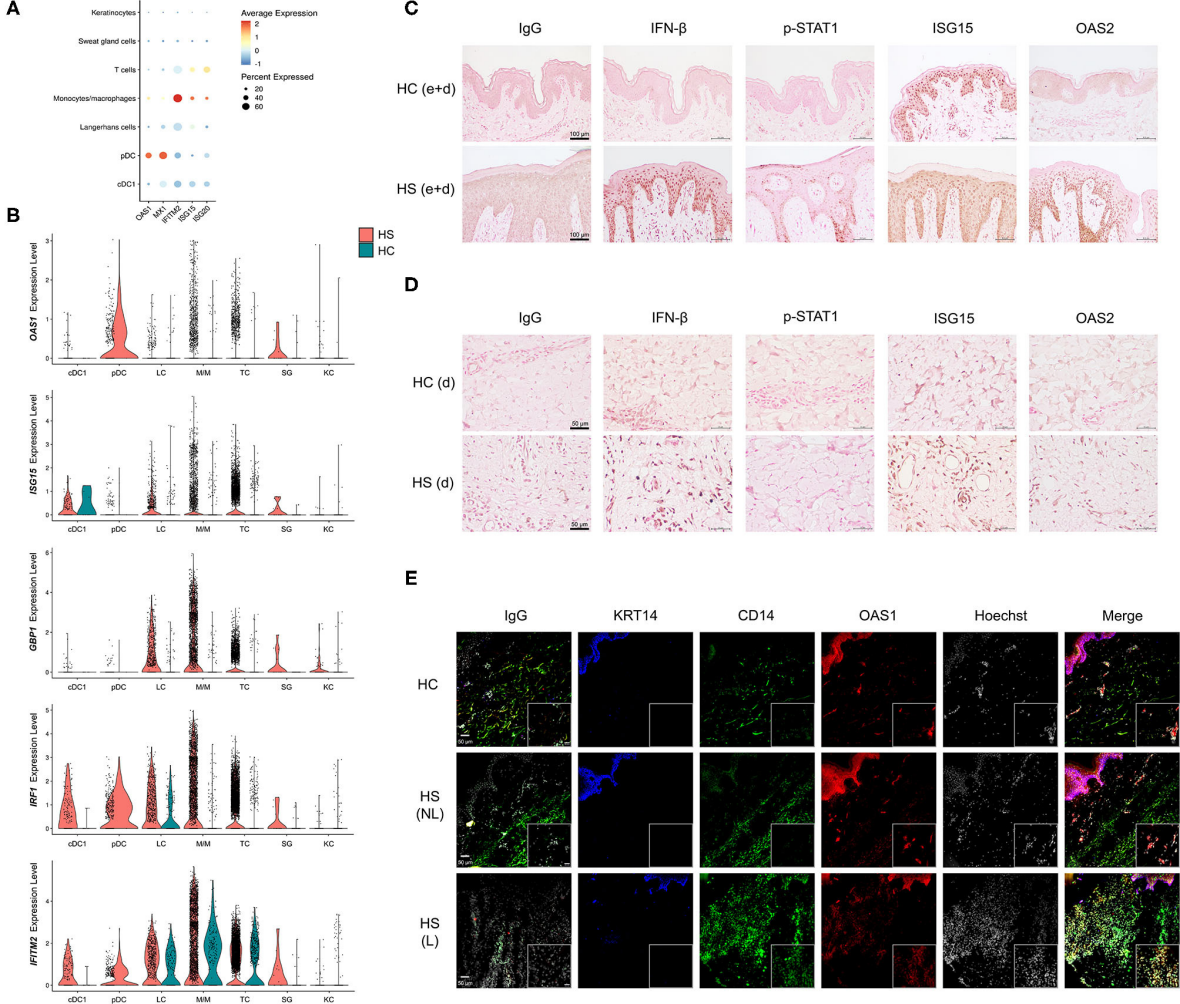
Key cell populations have increased expression and production of antiviral proteins within HS skin lesions. **(A)** Dot plot showing the percent expressed and average expression of antiviral proteins between key cellular populations in HS skin. **(B)** Violin plots of antiviral protein expression within key cellular populations within HS and HC skin. **(C,D)** Immunohistochemical staining of IFN-β, phospho (p)-STAT1, ISG15, and OAS2 in HS and HC skin with focus on **(C)** epidermis and dermis (e+d) at 20X (scale bar 100 μm) and **(D)** dermis (d) at 40X (scale bar 50 μm). **(E)** Immunofluorescence colocalization staining of CD14 (green) and OAS1 (red) at 20X. Scale bar for the 20X image is 50 μm, whereas, for the zoomed image is 20 μm. KRT (keratin)-14 (blue) staining visualizes the epidermis. cDC, conventional dendritic cell; HC, healthy control; KC, keratinocyte; LC, Langerhans cell; M/M, monocyte/macrophage; pDC, plasmacytoid dendritic cell; SG, sweat gland cell; TC, T cell; NL, non-lesional; L, lesional.

Among cell clusters identified from scRNA-seq analysis, we found that pDCs were among the highest in per-cell expression and percent total population expression of the AVPs *OAS1* and *MX1* (Figure 6A). *GBP1, GBP5, IRF1*, and *IFITM2* were highly expressed in monocytes/macrophages (Figures 4A, 6A,B). When examining differential gene expression between HS and HC skin within cell clusters, many immune cells demonstrated broad upregulation of AVPs, especially monocytes/macrophages (Figure 6B).

To investigate the production of AVPs in HS tissue, we performed immunohistochemistry (IHC) staining of multiple AVPs in HS lesions and HC skin (Figures 6C,D). Indeed, antibody reactivity against specific AVPs was stronger and more abundant within the epidermis and dermis of HS skin samples compared to HC skin (Figures 6C,D and Supplementary Figure 3). Specifically, we found that the AVPs IFN-β, p-STAT1, and OAS2 demonstrated more prominent staining throughout the epidermis of HS lesions, especially in the stratum basale, compared to HC skin (Figure 6C). Though ISG15 was expressed in HC skin, HS lesions contained higher levels of this protein (Figure 6C). Within the dermis of HS skin, we observed increased IFN-β, ISG15, and OAS2 relative to HC skin (Figure 6D). Some of these proteins were also elevated in ductal cells and sebaceous glands of HS samples (Supplementary Figure 3) We determined that key cell populations from our scRNA-seq analysis were likely contributing to the increased AVP visualization from IHC staining by performing immunofluorescence (IF) on patient-matched HS lesions and non-lesional skin samples, as well as HC skin. Consistent with our data, we found that CD14 colocalizes, at least in part, with OAS1 within the dermis of HS skin (Figure 6E). This corroborates our sc-RNA seq data by showing that CD14-positive monocytes/macrophages express AVPs in HS lesions (Figure 6E). CD14 expression was also observed in the epidermis with co-expression of OAS1 (Figure 6E).

**Figure 7:**
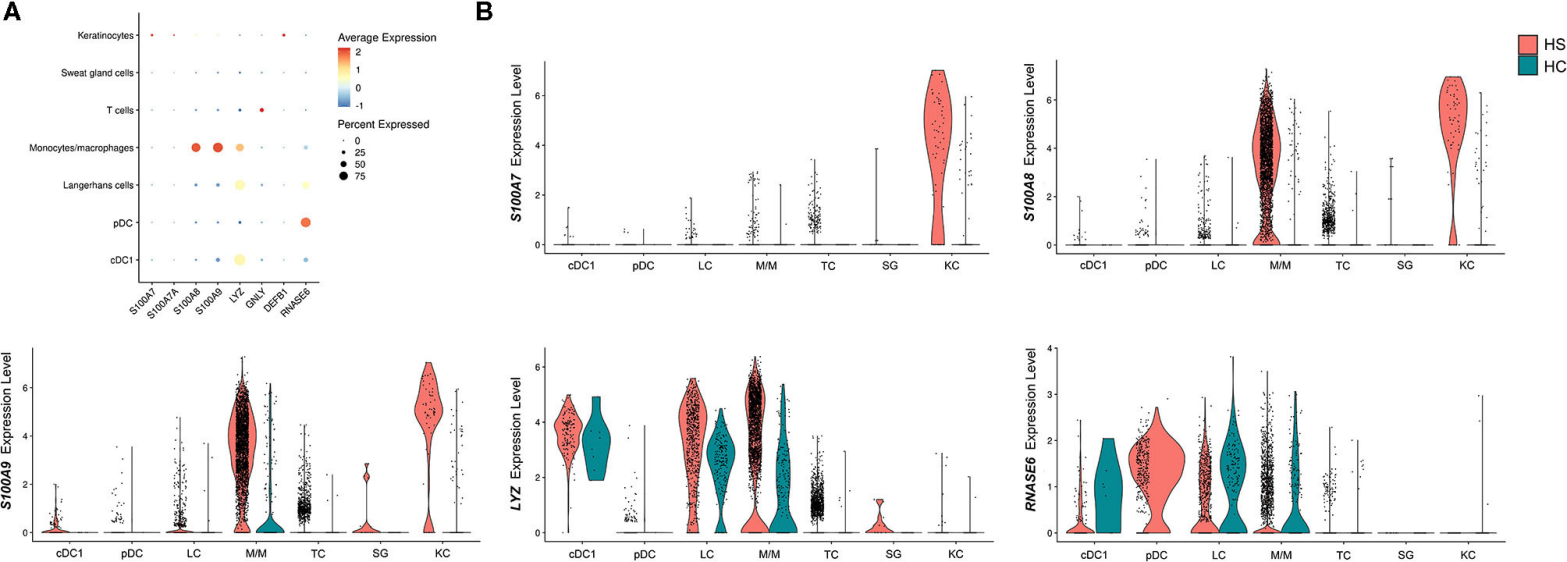
Antimicrobial peptides and proteins are transcriptionally upregulated in key cell populations within HS skin lesions. **(A)** Dot plot showing the percent expressed and average expression of AMPs between key cellular populations in HS skin. **(B)** Violin plots of AMP expression within key cellular populations in HS and HC skin. AMP, antimicrobial peptides and protein; cDC, conventional dendritic cell; HC, healthy control; KC, keratinocyte; LC, Langerhans cell; M/M, monocyte/macrophage; pDC, plasmacytoid dendritic cell; SG, sweat gland cell; TC, T cell.

Another important gene signature observed in the overlap analysis between HS, HWs, and DFUs was the upregulation of DEGs encoding for AMPs (Figure 1A). These molecules are important mediators of the innate immune response that inhibit microbial growth (83). In a previous study by our group, we found that the S100 family proteins S100A7, S100A7A, and S100A8 were transcriptionally upregulated in HS lesions compared to non-lesional skin (16). Within the same study, we found increased protein levels of S100A7 and S100A8 on IF of HS lesions compared to non-lesional skin (16). Therefore, we sought to characterize which cell types were contributing to the antimicrobial gene and protein signature in HS skin. When comparing AMP expression between different cell populations, we found that monocytes/macrophages and keratinocytes were the cells with the highest expression of *S100A8* and *S100A9* (Figures 7A,B). Compared to other cell populations, keratinocytes expressed the highest levels of *S100A7A* and *S100A7* (Figures 7A,B). Monocytes/macrophages, cDC1, and LCs had the highest expression of *LYZ*, which encodes for an enzyme that hydrolyzes glycosidic bonds in bacterial peptidoglycan (85) (Figures 7A,B). In addition to examining AMP expression across cell clusters, we also compared AMP expression between HS and HC skin on a per-cell basis. Keratinocytes in HS had increased expression of *S100A7* (log_2_FC = 3.82, *p* = 9.46E-63), *S100A7A* (log_2_FC = 1.55, *p* = 8.53E-49), *S100A8* (log_2_FC = 4.03, *p* = 2.68E-74), and *S100A9* (log_2_FC = 3.79, *p* = 4.96E-65) when compared with HC skin (Figure 7B). In monocytes/macrophages, *S100A8* (log_2_FC = 1.44, *p* = 1.03E-58), *S100A9* (log_2_FC = 1.09, *p* = 2.65E-61), as well as *LYZ* (log_2_FC = 1.42, *p* = 8.48E-21) were upregulated in HS samples (Figure 7B). *S100A7* (log_2_FC = 1.47, *p* = 4.39E-3), *S100A8* (log_2_FC = 1.73, *p* = 4.25E-4), and *S100A9* (log_2_FC = 1.25, *p* = 3.72E-6) were also upregulated within SG cells (Figure 7B).

In our overlap analysis, we found that DFUs and HS lesions have a shared downregulation of several SG-associated DEGs, such as *DCD, PIP*, and secretoglobins (Figure 1A). In fact, dermcidin (DCD), a SG-specific AMP, was one of the most downregulated DEGs in both the HS vs. non-lesional skin dataset (log2FC = −4.90, *p* = 2.01E-4) and DFU vs diabetic foot skin dataset (log_2_FC = −5.17, *p* = 1.27E-8) (30, 31). In contrast, in wounds that go on to heal, DCD was upregulated in the normal healing process (data not shown) (32). One limitation of microarray/bulk RNA-sequencing data is that it is not possible to identify if overall production of DCD in HS lesions and DFU is decreased and/or if tissue destruction due to inflammation results in loss of SG that produce DCD. With scRNA-seq, we were able to address this limitation. We found that fewer SGs were captured in the HS samples compared to HC and that expression of *DCD* in the SG cluster was not significantly decreased (log_2_FC = −0.361, *p* = 0.929) (Supplementary Figure 4). Since we here identified a possible reduction of SG in HS lesions, we analyzed our scRNA-seq data for other genes related to SG function. Interestingly, SG-associated DEGs between lesional and non-lesional skin that we had previously demonstrated to be downregulated in bulk transcriptomic analyses were actually upregulated in HS vs. HC skin in our scRNA-seq analysis (16) (Supplementary Figure 4). For instance, in the SG cell population, the secretoglobins *SCGB2A1* (log_2_FC = 0.671, *p* = 1.51E-3), *SCGB2B2* (log_2_FC = 0.257, *p* = 9.30E-3), and *SCGB3A1* (log_2_FC = 1.21, *p* = 4.92E-5) were upregulated (Supplementary Figure 4). One exception to these findings is *AQP5* (log_2_FC = −1.21, *p* = 2.79E-3), a gene involved in sweat production, that was downregulated in both the scRNA-seq and microarray datasets (Supplementary Figure 4). To examine the distribution of SG cells in HS skin, we performed IF on patient-matched HS lesions and non-lesional skin. Indeed, pan-keratin staining demonstrated apparent reduction of skin appendages, including SGs, in HS compared to HC skin (Supplementary Figure 5). Our data suggest that bulk transcriptomic reduction of SG-associated genes is associated with loss of SG structures in HS lesions rather than decreased expression of these genes.

## Discussion

In our study, we first analyzed the shared upregulated and downregulated DEGs of microarray/RNA-seq datasets from HS, HWs in the inflammatory phase, and DFUs. We found that HS lesions share important gene signatures and biological processes with chronic non-healing wounds, most notably those involved in macrophage function and antimicrobial/antiviral responses. Next, we performed scRNA-seq of several HS lesions. Because of the notable gene signatures from our overlap analysis, we focused our investigation of scRNA-seq data on monocytes and macrophages. We identified that this cellular population has increased expression of genes involved in phagocytosis, respiratory burst, and ADCC. A notable transcriptomic signature we observed was the upregulation of genes suggestive of an M1-like phenotype, which we validated *via* qPCR on HS tissue. Monocytes/macrophages also had higher expression and production of AVPs, as we demonstrated *via* IHC co-staining, and AMPs. Together, we show that macrophages may be key components of HS pathogenesis and targets for therapy.

It is known that M1 macrophages serve a pro-inflammatory role in the skin, performing phagocytosis and removal of cellular debris, while M2 macrophages contribute to wound repair and resolution of skin inflammation through secretion of anti-inflammatory cytokines and remodeling of the extracellular matrix and vasculature (14). In DFUs, a longer duration of the M1 phenotype leads to a chronic pro-inflammatory response and lack of wound healing (86). Thus, our scRNA-seq data demonstrate similarities between late-stage HS and chronic, non-healing wounds since monocytes/macrophages in both diseases are transcriptionally polarized toward an M1-like phenotype. Our data support previous scRNA-seq performed by Lowe and colleagues, who found decreased abundance of CD163-expressing macrophages in active inflammatory HS lesions (50). As previously described, CD163 is a characteristic marker of anti-inflammatory M2 macrophages (11). Notably, the authors also observed decreased abundance of CD163-expressing macrophages in non-lesional skin adjacent to HS lesions (50). Since non-lesional skin may be predisposed to developing HS pathology, further research is needed to clarify the role of macrophages in the transition and pathogenesis from non-lesional skin into lesional skin (87).

We identified that molecules within the FcR signaling pathway are upregulated in the monocyte/macrophage clusters. Given the many downstream effects of FcR activation, identifying the sources of this activation may help limit aberrant macrophage activity. Recent studies have shown a prominent B cell and plasma cell signature in HS lesions with increased immunoglobulin production (23, 50). Together with our data, this may indicate significant cross-talk between B cells/plasma cells and monocytes/macrophages in HS *via* immunoglobulin binding to FcR. Interestingly, Lowe and colleagues found that patients with greater B cell signatures and increased immunoglobulin production are less responsive to treatment with the anti-TNF-α therapy adalimumab (50). Because macrophages are a predominant source of TNF-α, increased immunoglobulin binding to Fc receptors may stimulate excess macrophage activation and cytokine release, contributing to the treatment resistance observed. Alternatively, FcR binding to therapeutic antibody may confer a therapeutic resistance mechanism. More studies are needed to further characterize these phenomena and to provide a better understanding as to why HS patients respond so heterogeneously to therapy; this may uncover paths to potentially stratify patients based on likelihood of clinical response. Furthermore, the upregulated FcR signaling pathway may also present a unique target for therapy, such as against the FcγRI receptor (CD64). Immunotherapy targeting CD64 for chronic wounds decreases the production of pro-inflammatory cytokines by reducing the predominance of M1 macrophages (55).

A significant gene signature that we uncovered in the overlap analyses between HS/DFU and HS/DFU/HW is an increase in IFN signaling and antiviral immunity. Though the relationship between cutaneous wound healing and antiviral defense remains poorly understood, Yang and colleagues as well as current ongoing work from our laboratory found that wounded tissue induces AVP expression and epithelial cell proliferation in an IL-27-dependent manner (88). However, the significance of this pathway in HS skin is still unclear as we did not identify a consistently significant transcriptional change in IL-27 in our scRNA-seq analysis. When examining the expression of IFN-related and antiviral genes on a per-cell basis, we identified that many cell populations have increased expression of these genes in HS lesions. The augmentation of immune cells within HS skin, especially monocytes/macrophages, may be mostly responsible for the increased production of AVPs and IFN-signaling molecules observed in IHC and IF staining. Interestingly, many IFN-stimulated and IFN signaling-associated molecules stained strongly within the epidermis on IHC and IF, although keratinocytes did not demonstrate the highest expression intensity or frequency of AVPs amongst all analyzed cell types. This could be explained by differences in transcriptional and protein detection and by the fact that protein accumulation could be higher in the epidermis due to the proximity and number of keratinocytes, even though the per-cell expression was low.

Whether the AVP and IFN signature is a primary or secondary process in HS remains unclear. Canonically, type I and type II IFNs participate in antiviral immunity by stimulating cellular and protein responses to viral infection (20). Both type I and type II IFNs are also involved in antitumor immunity (89, 90). Thus, it is plausible that the prominent IFN signature observed in chronic HS lesions is a response to viral or (pre)malignant processes, although therapy against microbial invasion in HS is generally directed toward bacteria (1). Whether an “antitumor” IFN response may be aimed toward eliminating (pre)malignant cells and the development of cutaneous squamous cell carcinoma or other cancers associated with HS is not fully understood (91). On the other hand, changes in the IFN gene signature may be secondary to dysregulated immune responses from ongoing cytotoxicity and the sensing of nucleic acid material from dying cells (92). pDCs are one of the most potent sources of type I IFNs and respond potently to alarmins, including danger-associated molecular patterns and pathogen-associated molecular patterns (80). Byrd and colleagues showed that pDCs within HS lesions colocalize with neutrophil extracellular traps (NETs) (26). Given that NETs stimulate production of IFN-α from pDCs, the upregulation of type I IFN-related genes may be secondary to dysregulated neutrophil activity, although antiviral NETosis has also been described (93, 94). Studies that explore the treatment of HS with therapies targeting type I and type II IFNs are not yet underway, but may be beneficial in elucidating the role of these pathways in disease pathogenesis.

In our scRNA-seq analysis, we identified T cells within HS skin lesions. While many studies have characterized T cell involvement in HS pathogenesis, a better understanding of the contributors to pathogenic T cell responses can guide therapeutic investigation for HS (50, 87, 95). Considering the reciprocal stimulatory interactions between macrophages and T cells, our study highlights macrophages as a potential therapeutic target to limit aberrant type 1 immune responses.

The inclusion of the DFU dataset to the HS/HW overlap analysis yields an antimicrobial gene signature similar to our previous study that compared HS and HW alone (16). On a per-cell basis, multiple immune and skin-structural cell populations have increased transcription of AMPs in HS lesions. In normal healing skin, AMPs protect from microbial infections and perform effector functions important for wound repair and reepithelialization (96). Dysregulation of AMPs has been implicated in abnormal wound healing (96). As members of the epidermal differentiation complex, the increased expression of S100 family proteins in HS lesions may lead to enhanced keratinocyte terminal differentiation and cornification and reduced regenerative ability (16, 97). Singh et al. (98) found that expression of toll-like receptor 9 and its downstream effector S100A8 is increased in chronic diabetic wounds. We hope that our data can pave the way for future studies to elucidate which AMPs produced by what cell populations are most important for wound healing in HS.

Collective transcriptional changes of M1 macrophage polarization and function, enhanced NK cell and T cell cytotoxicity, and an IFN gene signature at a cellular level indicate an enriched type 1 immune response within HS lesions. Type 1 effector immunity involves cellular responses to intracellular pathogens such as bacteria and viruses (70). Interestingly, the microbiome is significantly altered in patients with late-stage HS such that gram-negative anaerobes predominate in suppurating lesions and sinus tracts (1). Given that our excisional samples were collected from patients with late-stage HS, the observed type 1 immune profile may indicate a response to intracellular bacterial growth. Bacteria frequently isolated from late-stage HS such as *Porphyromonas* and *Fusobacterium* are capable of switching from extracellular to intracellular replication in epithelial cells in an actin-dependent process (99, 100). Since the role of microbial changes in HS pathogenesis is poorly understood, future studies should explore the significance of this potential transition.

This study had several limitations. First, we did not identify the presence of neutrophils in our scRNA-seq analysis, even though these cells are known to infiltrate HS skin (50, 101). This may be explained by the fact that our skin dissociation protocol for scRNA-seq analysis involved gradient centrifugation of the skin cell suspensions, which removed cellular debris, as well as erythrocyte and neutrophil populations. Nevertheless, neutrophils play an important role in HS pathogenesis, promoting release of the proinflammatory cytokine IL-17 and the proinflammatory AMPs S100A8 and S100A9 (101). In-depth single-cell analysis of neutrophils and their pathways in ongoing and future studies may further elucidate disease mechanisms and areas of therapeutic exploration (23, 50). Our study was also limited by small sample sizes for HS and HC skin and lacked patient-matched peri-lesional and non-lesional skin. Additionally, the de-identified HS samples that we used for the scRNA-seq analysis were collected from surgical excisions of patients; this procedure is typically only indicated for patients with moderate to severe HS (102). Thus, the data may only reflect the transcriptional profile of chronic HS marked by cycles of inflammatory damage and structural distortion, which may narrow the generalizability of our results to a subset of HS patients, in particular those with severe disease.

## Conclusion

Hidradenitis suppurativa is a chronic inflammatory skin disorder that frequently impairs quality of life. Open HS lesions and sinus tracts characteristic of late-stage disease share features with chronic non-healing wounds such as DFUs and chronic venous leg ulcers. Here, we investigated the transcriptional and cellular landscape of HS lesions to better understand the immune processes and pathways that may underlie disease pathogenesis.

Transcriptome analysis of shared DEGs between HS lesions and DFUs reveals increased expression of AMPs, IFN-related proteins, and molecules involved in phagocytosis and FcR-mediated signaling. These gene signatures are reflected on a per-cell basis in key cell populations as revealed by scRNA-seq of HS lesions. On the basis of key transcriptional changes, monocytes/macrophages are polarized toward an M1 macrophage phenotype with increased phagocytosis, FcR signaling, respiratory burst, and execution of ADCC. These macrophage pathways are potential targets for therapy, such as the CD64 Fcγ receptor. Antiviral immunity is augmented across multiple cell types; importantly, many genes involved in type I and type II IFN responses are upregulated in monocytes/macrophages and their protein products can be visualized *via* IHC and IF. Antimicrobial immunity is also enhanced across various cell populations. Overall, we show that IFN signaling and macrophages contribute to the dysregulated expression of proinflammatory genes and pathways in HS lesions.

## Data Availability Statement

The datasets presented in this study can be found in online repositories. The names of the repository/repositories and accession number(s) can be found below: GSE175990.

## Ethics Statement

The studies involving human participants were reviewed and approved by Duke University Health System Institutional Review Board and UT Southwestern Institutional Review Board. The patients/participants provided their written informed consent to participate in this study.

## Author Contributions

PM and AM contributed to the overall conception and design of the study. AM directed the overall study and supervised all aspects of the study and writing. PM, SJ, CP, MC, DB, DE, TJ, and TH-T collected tissue and/or data. PM, SJ, CP, JS, MW, TH-T, and AM contributed to the interpretation of the results. VJ organized the single-cell sequencing database. VJ, SG, and DC performed statistical and biocomputational analyses. PM, SJ, and AM wrote the first draft of the manuscript. JS wrote sections of the manuscript. All authors contributed to manuscript edits, revision, and read and approved the submitted version.

## Conflict of Interest

AM consulted for Silab and received a project grant from Silab to support her lab. Silab did not have any insight into the current data and had no decision in publishing or ownership. AM served as a SEC member of the LEO Foundation in the recent past and is currently employed by Janssen. The spouse of AM was employed by Precision Biosciences and holds stock and stock options. The remaining authors declare that the research was conducted in the absence of any commercial or financial relationships that could be construed as a potential conflict of interest.

## Publisher's Note

All claims expressed in this article are solely those of the authors and do not necessarily represent those of their affiliated organizations, or those of the publisher, the editors and the reviewers. Any product that may be evaluated in this article, or claim that may be made by its manufacturer, is not guaranteed or endorsed by the publisher.

## Abbreviations

HS: hidradenitis suppurativa
IFN: interferon
DEG: differentially expressed gene
DFU: diabetic foot ulcer
SG: sweat gland
TNF: tumor necrosis factor
IL: interleukin
LPS: lipopolysaccharide
AMP: antimicrobial peptide and protein
AVP: antiviral protein
hBD: human β-defensin
OAS: oligoadenylate synthase
HW: healing wound
scRNA-seq: single-cell RNA sequencing
ADCC: antibody-dependent cellular cytotoxicity
pDC: plasmacytoid dendritic cell
Th: T-helper cell
IRB: Institutional Review Board
GEMS: gel beads in emulsion
UMAP: uniform manifold approximation and projection
ISG: interferon-stimulated gene
GOrilla: gene ontology enrichment analysis (and visualization tool)
DC: dendritic cell
LC: Langerhans cell
FcR: Fc receptor
ROS: reactive oxygen species
SOD: superoxide dismutase
NK: natural killer
STAT: signal transducer and activator of transcription
GAS: γ-interferon activated sequence
ISRE: interferon-sensitive response element
cDC: Classical dendritic cell
IHC: immunohistochemistry
IF: immunofluorescence
DCD: dermcidin
NET: neutrophil extracellular trap.
