# Supplementary material for: Single-Cell RNA Sequencing Reveals Cellular and Transcriptional Changes Associated With M1 Macrophage Polarization in Hidradenitis Suppurativa
Source: Front Med (Lausanne). 2021 Aug 24;8:665873. doi: 10.3389/fmed.2021.665873 (PMC8421606; doi:10.3389/fmed.2021.665873)
Supplement: Supplementary file 1 [file Data_Sheet_1.DOCX]

**Supplementary Table 1. Clinical characteristics of patients included in single-cell RNA sequencing.**

| **Patient** | **Sex** | **Age** | **Race** | **Hurley stage** | **Location** |
| --- | --- | --- | --- | --- | --- |
| HS 1 | Female | 35 | African-American | 3 | Axilla |
| HS 2 | Female | 27 | African-American | 3 | Axilla |
| HS 3 | Female | 66 | African-American | 3 | Axilla |
| HC 1 | Male | 48 | African-American | - | Abdomen |

**Supplementary Table 2. Primer sequences and their melting temperatures for qPCR.**

| **Primer** | **Sequence** | **Tm (°C)** |
| --- | --- | --- |
| hGAPDH F | ATGGGAAGGTGAAGGTCGGA | 58.2 |
| hGAPDH R | CAGCGTCAAAGGTGGAGGAGT | 59.4 |
| hGBP1 F | CCAGTTGCTGAAAGAGCAAGAGA | 57.3 |
| hGBP1 R | TCCCTCTTTTAGTAGTTGCTCCTGTT | 57.5 |
| hGBP5 F | TCACTTCAAGGTGCCCATATC | 54.7 |
| hGBP5 R | CATTCTCCCTGCTTCCTCATAG | 54.8 |
| hIRF1 F | GGAAGTCGTGGAGATGCTATAC | 54.7 |
| hIRF1 R | GACAGAAGAGCAAGAGGCTATC | 54.9 |
| hSTAT1 F | ACCGCACCTTCAGTCTTTTCC | 57.7 |
| hSTAT1 R | TGAACTGGACCCCTGTCTTCA | 58.0 |

**Supplementary Table 3. Signature genes used to define cluster identity.**

| **Cluster** | **Cell type** | **Genes** |
| --- | --- | --- |
| 0 | Endothelial cells | *CD34, ICA1, PECAM1, SELE, VWF* |
| 1 | B cells | *CD19, CD22, CD27, CD79A, CD79B* |
| 2 | Plasma cells | *CD27, CD38, CD79A, SDC1 (CD138)* |
| 3 | T cells | *CD3D, CD3E, CD3G, CD4* |
| 4 | T cells | *CD3D, CD3E, CD3G, CD4* |
| 5 | Monocytes/macrophages | *CD14, FCGR1A (CD64), FCGR3B (CD16)* |
| 6 | Monocytes/macrophages | *CD11B, CD14, CD68, CD163, FCGR1A (CD64), FCGR3A (CD16), LYZ* |
| 7 | Fibroblasts | *CD81, COL1A1, DCN, LRP1, LUM, MME* |
| 8 | T cells | *CD3D, CD3E, CD3G, CD8A* |
| 9 | Plasma cells | *CD27, CD38, CD79A, SDC1 (CD138)* |
| 10 | Langerhans cells | *CD1A, CD1C, CD4, CD207* |
| 11 | Pericytes | *ACTA2, MCAM (CD146), NES, PDGFRB, RSG5* |
| 12 | Natural killer cells | *KLRD1 (CD94), NCAM1 (CD56), NCR1 (NKP46), NKG7* |
| 13 | Fibroblasts | *CD81, COL1A1, DCN, LRP1, LUM, VIM* |
| 14 | Fibroblasts | *CD81, COL1A1, DCN, ITGB1, LRP1, VIM* |
| 15 | Monocytes/macrophages | *CD11B, CD14, CD68, CD163, FCGR3A (CD16), LYZ* |
| 16 | Mitotic cells | *KI67, PCNA* |
| 17 | Keratinocytes | *DSG1, DSP, KRT5, KRT10, KRT14, KRT17, S100A2* |
| 18 | T cells | *CD3D, CD3E, CD3G, CD8A* |
| 19 | Erythrocytes | *HBA1, HBA2, HBB* |
| 20 | Plasma cells | *CD27, CD38, CD79A, SDC1 (CD138)* |
| 21 | T cells | *CD3D, CD3E, CD3G, CD8A* |
| 22 | T cells | *CD3D, CD3E, CD3G, CD4* |
| 23 | Plasmacytoid dendritic cells | *CD4, CLEC4C, FCER1G, IL3R (CD123), NRP1, TNFRSF21* |
| 24 | Melanocytes | *MLANA, PMEL, TYRP1* |
| 25 | Endothelial cells | *CD81, PECAM1, VIM, VWF* |
| 26 | Plasma cells | *CD27, CD38, CD79A, SDC1 (CD138)* |
| 27 | Mast cells | *ENPP3 (CD203C), FCER1A, KIT (CD117), TPSB2* |
| 28 | Conventional dendritic cells 1 | *BTLA, CADM1, CLEC9A, THBD* |
| 29 | Endothelial cells | *CD34, CD81, PECAM1, VIM, VWF* |
| 30 | Sweat gland cells | *AQP5, DCD, KRT8, KRT15, KRT18, PIP, SCGB1B2P, SCGB1D2* |
| 31 | Conventional dendritic cells 2 | *CD68, CLEC4A, CLEC7A, FCER1G, SIRPA* |

**Supplementary Table 4. Genes related to type 1 immunity and cellular activation are upregulated in Langerhans cells within HS lesions.** log_2_FC of gene expression was calculated by comparing HS samples to healthy skin within the LC clusters. FC = fold change; IFN = interferon; LC = Langerhans cell; Th1 = type 1 helper T cell

| **Gene** | **Protein class** | **log_2_FC** | **p-value** | **Function in LC** | **Ref.** |
| --- | --- | --- | --- | --- | --- |
| *CXCL9* | Chemokine | 1.45 | 4.25E-12 | Immune cell migration;  Th1 polarization | (1) |
| *CXCL10* | Chemokine | 0.661 | 2.04E-6 | Immune cell migration;  Th1 polarization | (1) |
| *STAT1* | Transcription factor | 0.995 | 1.60E-33 | IFN response and cellular maturation | (2) |
| *IL18* | Interleukin | 0.541 | 6.62E-13 | Induce IFN-γ secretion (IL-12-dependent) | (3) |
| *CD40* | Costimulatory protein | 0.441 | 1.81E-11 | Costimulation signal; prime T cells | (4) |
| *CD86* | Costimulatory protein | 0.292 | 2.82E-11 | Costimulation signal; prime T cells | (5) |
| *HLA-A* | MHC Class I | 0.805 | 1.02E-42 | Antigen presentation | (6) |
| *HLA-C* | MHC Class I | 1.34 | 2.22E-63 | Antigen presentation | (6) |
| *HLA-DQA2* | MHC Class II | 0.779 | 4.34E-8 | Antigen presentation | (6) |
| *HLA-DRB5* | MHC Class II | 1.63 | 2.23E-22 | Antigen presentation | (6) |
| *LAMP3* | Lysosome-associated membrane glycoprotein | 1.40 | 1.86E-22 | Activation and MHC Class II presentation | (7) |


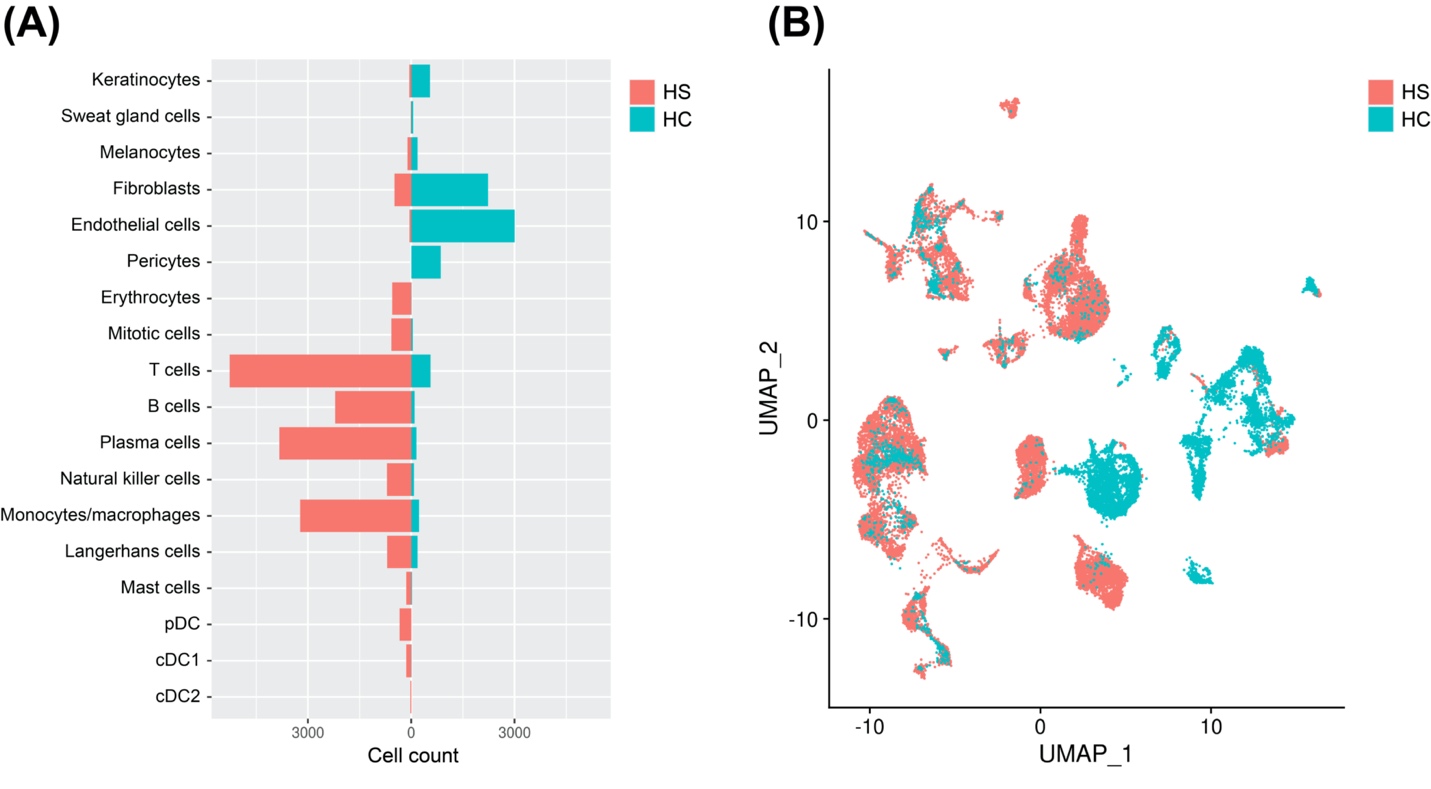


**Supplementary Figure 1. Distribution of cell clusters within HS and HC skin.** (A) Sample distribution of cell populations by cell count organized by whether they came from HS or HC skin. (B) UMAP plot of clusters organized by whether they came from HS or HC skin. cDC = conventional dendritic cell; HC = healthy control; pDC = plasmacytoid dendritic cell

**
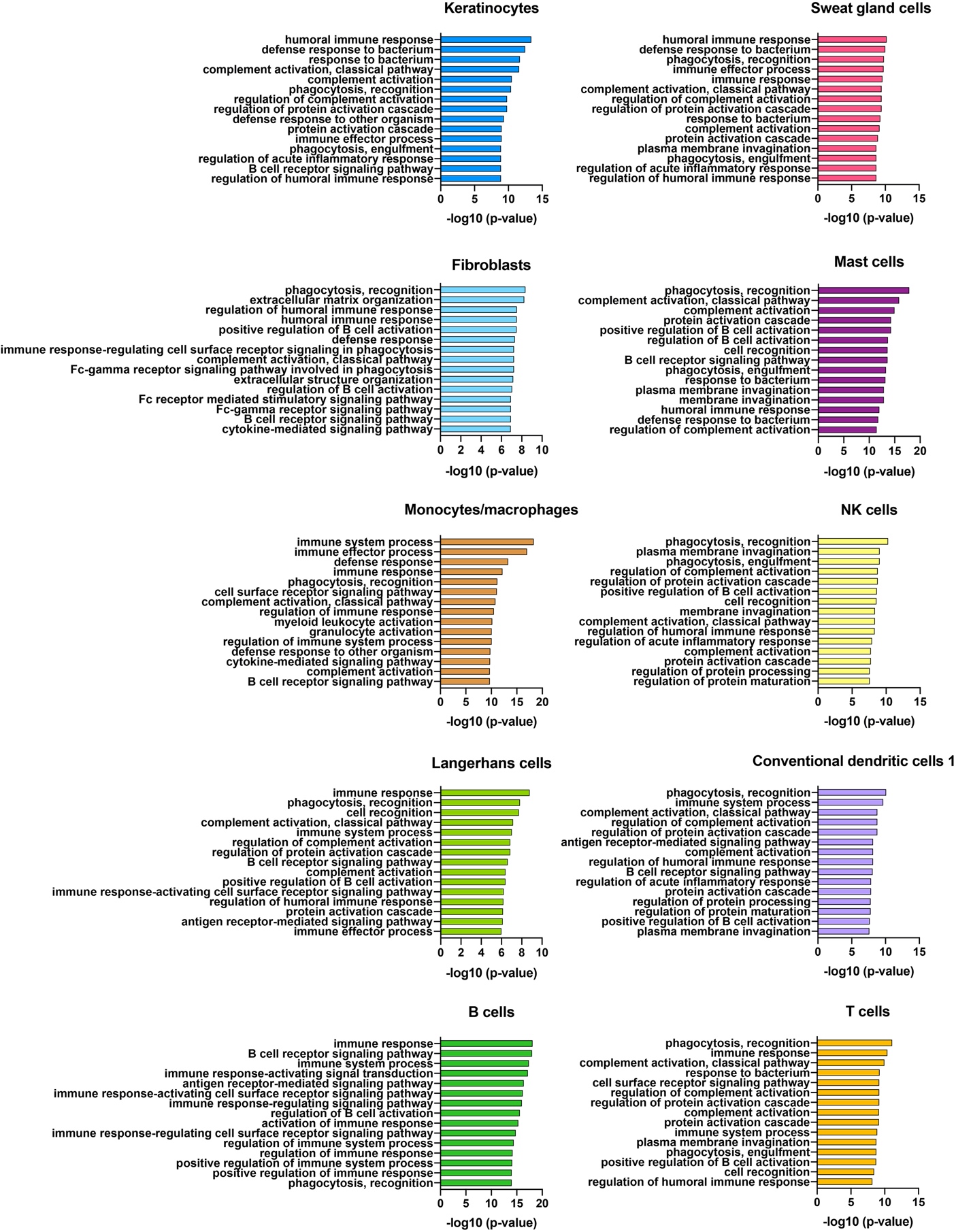
Supplementary Figure 2. Enriched biological processes among differentially-expressed genes between HS and healthy control skin.** Top 15 enriched GO terms with the lowest p-values are represented for select epithelial and immune cell clusters. p-values were calculated according to the minimum hypergeometric model.

**
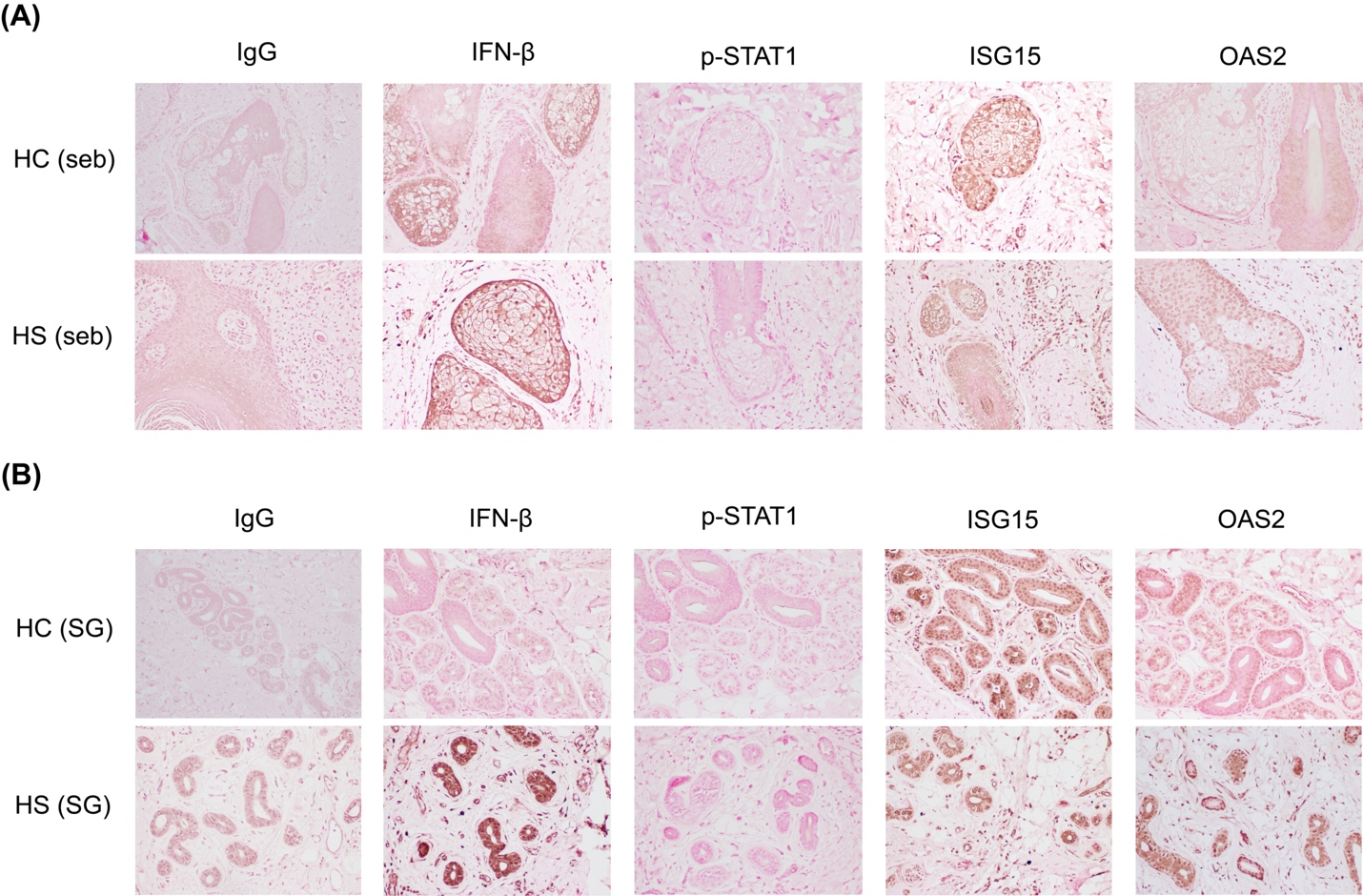
**

**Supplementary Figure 3. Immunohistochemistry reveals an AVP and IFN signature in HS skin.** IHC staining for IFN-β, phospho (p-)STAT1, ISG15, and OAS2 in HS and HC skin depicting (A) sebaceous gland (seb) and (B) sweat gland (SG) structures in dermal tissue samples at 20X magnification. AVP = antiviral protein; HC = healthy control; IFN = interferon; IHC = immunohistochemistry

**
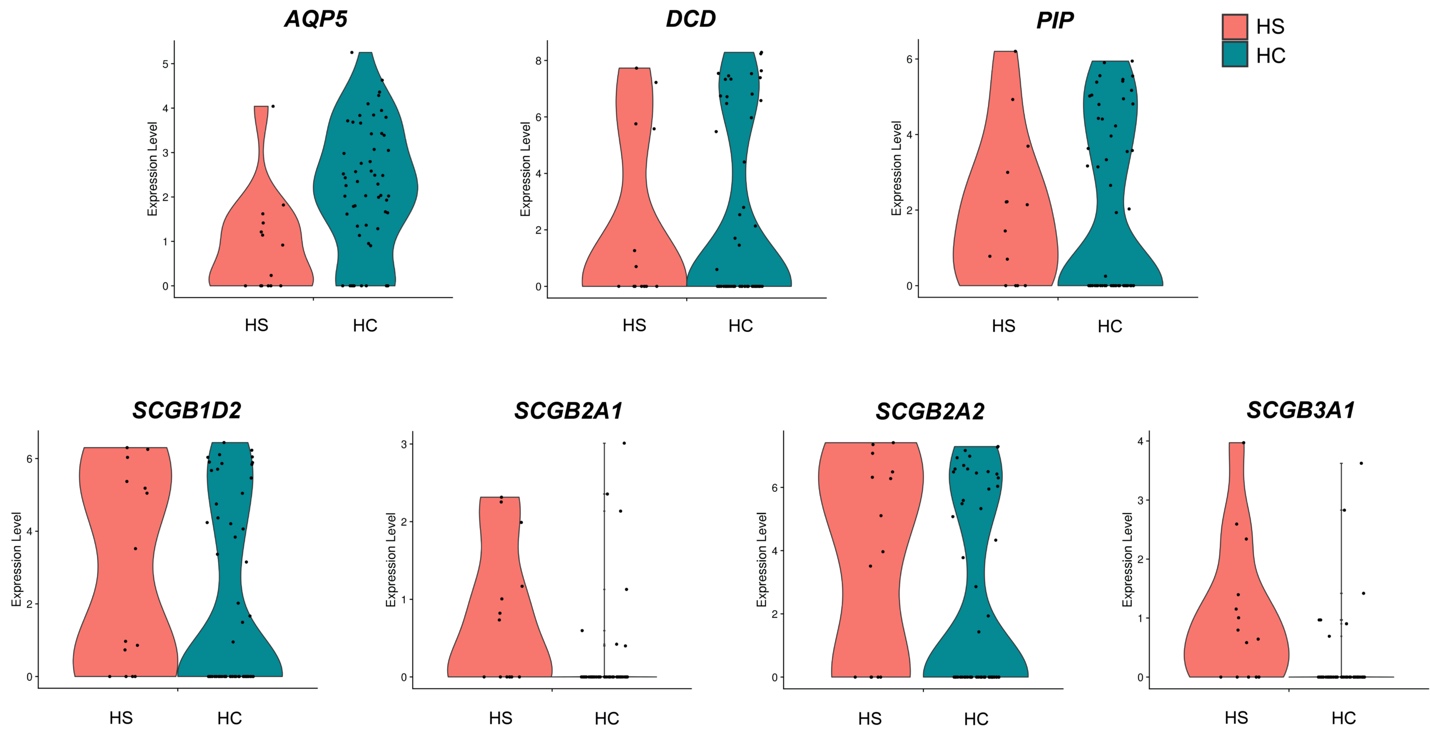
**

**Supplementary Figure 4. Sweat gland-associated genes are transcriptionally dysregulated in sweat gland cells within HS skin lesions.** Violin plots of sweat gland-associated gene expression within the sweat gland population in HS and healthy control (HC) skin.

**
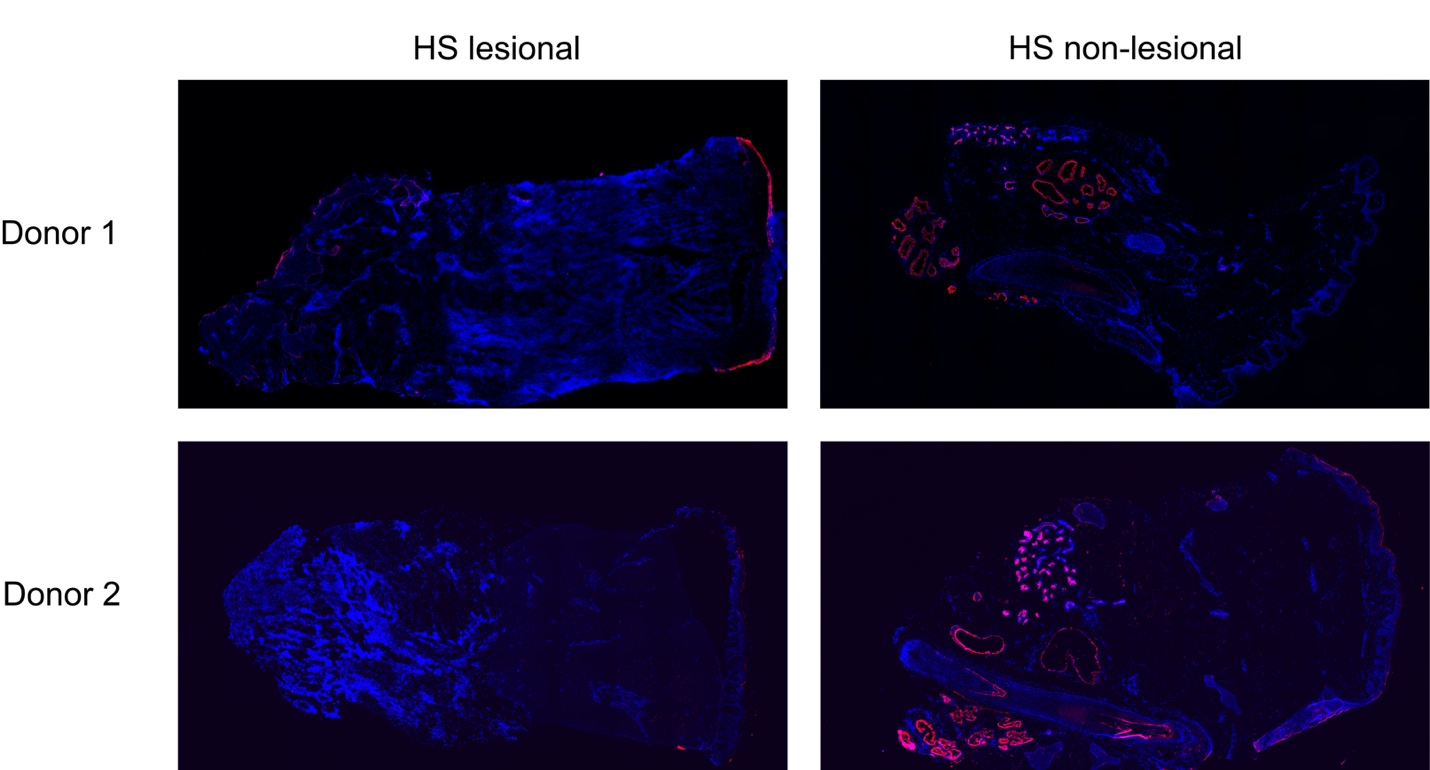
**

**Supplementary Figure 5. HS lesions demonstrate a loss of skin appendages compared to HC skin.** Pan-cytokeratin staining of whole tissue isolated from donor-matched HS lesional and non-lesional skin. HC = healthy control

**References**

1. Tokunaga R, Zhang W, Naseem M, Puccini A, Berger MD, Soni S, et al. CXCL9, CXCL10, CXCL11/CXCR3 axis for immune activation - A target for novel cancer therapy. Cancer Treat Rev [Internet]. 2017/11/26. 2018 Feb;63:40–7. Available from: https://pubmed.ncbi.nlm.nih.gov/29207310

2. Jackson SH, Yu C-R, Mahdi RM, Ebong S, Egwuagu CE. Dendritic Cell Maturation Requires STAT1 and Is under Feedback Regulation by Suppressors of Cytokine Signaling. J Immunol [Internet]. 2004 Feb 15;172(4):2307 LP – 2315. Available from: http://www.jimmunol.org/content/172/4/2307.abstract

3. Nakanishi K. Unique Action of Interleukin-18 on T Cells and Other Immune Cells [Internet]. Vol. 9, Frontiers in Immunology. 2018. p. 763. Available from: https://www.frontiersin.org/article/10.3389/fimmu.2018.00763

4. Gorbachev A V, Fairchild RL. CD40 Engagement Enhances Antigen-Presenting Langerhans Cell Priming of IFN-γ-Producing CD4&lt;sup&gt;+&lt;/sup&gt; and CD8&lt;sup&gt;+&lt;/sup&gt; T Cells Independently of IL-12. J Immunol [Internet]. 2004 Aug 15;173(4):2443 LP – 2452. Available from: http://www.jimmunol.org/content/173/4/2443.abstract

5. Rattis FM, Péguet-Navarro J, Staquet MJ, Dezutter-Dambuyant C, Courtellemont P, Redziniak G, et al. Expression and function of B7-1 (CD80) and B7-2 (CD86) on human epidermal Langerhans cells. Eur J Immunol. 1996 Feb;26(2):449–53.

6. Wieczorek M, Abualrous ET, Sticht J, Álvaro-Benito M, Stolzenberg S, Noé F, et al. Major Histocompatibility Complex (MHC) Class I and MHC Class II Proteins: Conformational Plasticity in Antigen Presentation. Front Immunol [Internet]. 2017 Mar 17;8:292. Available from: https://pubmed.ncbi.nlm.nih.gov/28367149

7. Malaguarnera L, Marsullo A, Zorena K, Musumeci G, Di Rosa M. Vitamin D3 regulates LAMP3 expression in monocyte derived dendritic cells. Cell Immunol [Internet]. 2017;311:13–21. Available from: https://www.sciencedirect.com/science/article/pii/S0008874916301137
